# Plasma-based proteomic profiling identifies the distinct regulation of proteins in hyperplasia and endometrial cancer

**DOI:** 10.1186/s12885-024-12522-0

**Published:** 2024-06-20

**Authors:** Khalid Akkour, Ibrahim O Alanazi, Assim A Alfadda, Afshan Masood, Hani Alhalal, Salini Scaria Joy, Ali Bassi, Eman Alshehri, Moudi A Alwehaibi, Maria Arafah, Hicham Benabdelkamel

**Affiliations:** 1 Obstetrics and Gynecology Department, College of Medicine, King Saud University, Riyadh, 11461 Saudi Arabia; 2 Healthy Aging Research Institute, King Abdulaziz City for Science and Technology (KACST), Health Sector, Riyadh, 11442 Saudi Arabia; 3 Proteomics Resource Unit, Obesity Research Center, College of Medicine, King Saud University, Riyadh, 11461 Saudi Arabia; 4 Department of Medicine, College of Medicine, King Saud University, Riyadh, 11461 Saudi Arabia; 5 Strategic Center for Diabetes Research, College of Medicine, King Saud University, Riyadh, 11461 Saudi Arabia; 6 Department of Pathology, College of Medicine, King Saud University, King Saud University Medical City, Riyadh, 11461 Saudi Arabia

**Keywords:** Endometrial cancer, Hyperplasia, Plasma, Proteomics, 2D-DIGE, MALDI-TOF, Biomarker, Vitamin D binding protein

## Abstract

**Background:**

Among gynaecological malignancies, endometrial cancer (EC) is the most prevalent type of uterine cancer affecting women. This study explored the proteomic profiles of plasma samples obtained from EC patients, those with hyperplasia (Hy), and a control group (CO). A combination of techniques, such as 2D-DIGE, mass spectrometry, and bioinformatics, including pathway analysis, was used to identify proteins with modified expression levels, biomarkers and their associated metabolic pathways in these groups.

**Methods:**

Thirty-four patients, categorized into three groups—10 with EC, 12 with Hy, and 12 CO—between the ages of 46 and 75 years old were included in the study. Untargeted proteomic analysis was carried out using two-dimensional difference in gel electrophoresis (2D-DIGE) coupled with matrix-assisted laser desorption/ionization time-of-flight mass spectrometry (MALDI-TOF-MS).

**Results:**

In all three groups, 114 proteins that were significantly (*p* ≤ 0.05 and fold change ≥ 1.5) altered were successfully identified using peptide mass fingerprints (PMFs). Compared with those in the control group (CO), the EC samples had 85 differentially expressed proteins (39 upregulated and 46 downregulated), and in the Hy group, 81 proteins were dysregulated (40 upregulated and 41 downregulated) compared to those in the CO group, while 33 proteins exhibited differential regulation (12 upregulated and 21 downregulated) in the EC plasma samples compared to those in the Hy group. Vitamin D binding protein and complement C3 distinguished Hy and EC from CO with the greatest changes in expression. Among the differentially expressed proteins identified, enzymes with catalytic activity represented the largest group (42.9%). In terms of biological processes, most of the proteins were involved in cellular processes (28.8%), followed by metabolic processes (16.7%). STRING analysis for protein interactions revealed that the significantly differentially abundant proteins in the three groups are involved in three main biological processes: signalling of complement and coagulation cascades, regulation of insulin-like growth factor (IGF) transport and uptake by insulin-like growth factor binding proteins (IGFBPs), and plasma lipoprotein assembly, remodelling, and clearance.

**Conclusion:**

The identified plasma protein markers have the potential to serve as biomarkers for differentiating between EC and Hy, as well as for early diagnosis and monitoring of cancer progression.

**Supplementary Information:**

The online version contains supplementary material available at 10.1186/s12885-024-12522-0.

## Introduction

Endometrial cancer (EC) is considered the most common gynaecologic malignancy in high-income countries, with an alarming increase in its incidence globally [1]. In the last 30 years, the incidence has increased by 132%, particularly in individuals with obesity and the ageing population [2]. According to reports from the GLOBOCAN series of the International Agency for Research on Cancer, the estimated incidence of corpus uteri cancer in females of all ages was 417,367 (4.5%), the mortality rate was 97,370, and the number of prevalent cases was 1,415,213 globally in 2020. Corpus uteri cancer is the 4th most common cancer in Saudi females, with an incidence of 1,016 (7.5%), an estimated mortality of 293 (5.5%) and a prevalence of 3,679 (8.5%) in the same year [3]. EC is also called corpus uteri cancer as it arises from the epithelial lining of the uterine cavity [4]. EC can be classified into histological subtypes and molecular phenotypes [5]. Historically, EC has been categorized into endometrioid (type I) and nonendometrioid (type II) [6]. Type I affects approximately 80% of patients [6] and is associated with unopposed oestrogen stimulation, low grade, a favourable prognosis and a mutation in phosphate and tensin homologue (PTEN). Type I ECs are composed of grade I or II endometrioid adenocarcinomas [7]. Type II disease affects approximately 20% of patients and is non-oestrogen dependent, with high rates of metastasis, high grade, high aggressiveness and an unfavourable prognosis [7–9]. Type II commonly occurs in aged patients who have mutations in a tumour suppressor gene (P53) [10, 11]. Type II ECs are composed of Grade III endometrioid adenocarcinomas, undifferentiated, carcinosarcomas and serous clear cells [7]. Molecular classification by the Cancer Genome Atlas (TCGA) Research Network led to the identification of 4 molecular subgroups of EC. These subgroups were defined by copy number alterations and mutation burden through the study of 373 patients with EC in the TCGA [12]. Early diagnosis is key to improving survival. Therefore, several diagnostic procedures, such as endometrial biopsy, hysteroscopy and transvaginal ultrasound, have been utilized for diagnosing EC. However, due to the low specificity, invasiveness, cost and difficulty in performing these procedures, there is an urgent need for specific biomarkers for better diagnosis [13]. Since the proteome is reflects the dynamic state of cells, tissues and organisms, proteomics has the potential to identify relevant biomarkers for cancer diagnosis [14].

Proteomic technologies in combination with computational analyses appear to be potent tools for identifying potential biomarkers [15]. According to a study by Lee et al., women with EC and a p53 mutation were approximately 11 times (95% CI: 1.01–120.7) more likely to die from the disease than women without the mutation [16]. Chen et al. reported that a high level of vascular endothelial growth factor (VEGF) in the cytoplasm is common in stage II EC and above, and that a level above 800 pg/mg is a risk factor for cancer recurrence [17]. PTEN is the most common gene mutated in EC, occurring in 25–83% of all cases. Therefore, PTEN mutation is considered an early event in EC development [18]. Moreover, a number of candidate EC biomarkers have been identified, including CA15-3, CA19-9, CA-125, and HE4 for diagnosis and Survivin, L1CAM, c-erb B2, and COX-2 for prognosis. Due to their lack of specificity and sensitivity, they have limited significance in clinical practice [19]. In our previous study, tissue-based proteomic profiling of EC and hyperplasia patients revealed that the expression of proteins, including peptidyl prolyl cis-trans isomerase A, zinc finger protein, fructose bisphosphate aldolase A, and alpha enolase, among others, was significantly altered between groups, with *p* ≤ 0.05 and a fold change ≥ 1.5 [20]. The inherent variability of tissue samples poses a significant challenge to accurately interpreting laboratory results. This variability can stem from differences in the composition and properties of tissues, making it difficult to draw definitive conclusions from experimental data. As a result, there is an urgent need for specific and sensitive biomarkers for diagnosing EC. Several articles have indicated that two-dimensional difference gel electrophoresis (2D-DIGE) coupled with mass spectrometry (MS) offers significant advantages in exploring a wide range of biological samples to identify cancer biomarkers, including ovarian cancer, gastric cancer, liver cancer and EC [21–26]. In the present study, 2D-DIGE coupled with MS was used to study the proteomic profile of depleted serum among the EC, Hy, and CO groups of Saudi females to identify novel biomarkers for early screening, diagnosis and progression of EC.

## Materials and methods

### Ethical approval and consent to participate

The study procedures and protocols were reviewed and approved by the institutional review board of the College of Medicine, King Saud University. Written informed consent was obtained from all the participants (IRB number: E-193,622). This study was conducted at the Proteomics Resource Unit, Obesity Research Center, College of Medicine, and King Khalid University Hospital (KKUH), King Saud University, Riyadh, Saudi Arabia.

### Study design and patient selection

Patients aged 46–75 years old who were admitted to the outpatient clinics of the Obstetrics and Gynaecology-Oncology Department, King Khalid University Hospital, College of Medicine, King Saud University, were recruited for the study. A total of 34 women were included. The primary assessment was performed during clinical appointments. Patients willing to participate in the study were recruited, and informed consent was obtained. The patients were divided into endometrial cancer (EC), hyperplasia (Hy), and control (CO) groups. EC and Hy were confirmed based on histopathological examination. EC patients (*n* = 10): women diagnosed with EC. Hy patients (*n* = 12): Women diagnosed with hyperplasia; CO individuals (*n* = 12): healthy women. The study excluded all women with other types of cancer, active inflammatory disease or other autoimmune diseases. Stage, grade of the cancer, and anthropometric measures, including height and weight, were assessed in the clinics while the subject was wearing light clothing, and body mass index (BMI) was calculated as body weight divided by height squared (kg/m2). The characteristics of the study participants are shown in Table S1; Additional File 1.

The sample size was determined by conducting a power analysis using Progenesis SameSpots nonlinear dynamics statistical software to determine the minimum number of biological replicates needed. Blood samples were drawn from all 34 patients, including patients with EC, Hy, and CO. EDTA-containing tubes were used to collect blood samples. Plasma was prepared by centrifugation at 2,500× g for 20 min at 4 °C. Afterwards, the plasma samples were aliquoted and stored at − 80 °C until further use.

### Depletion of abundant proteins

Prior to any proteomics experiments, plasma samples were carefully processed to deplete highly abundant proteins, such as albumin, immunoglobulins, alpha-1 antitrypsin and transferrin that may interfere with MS analysis and biomarker detection. Depletion was performed using a multiple affinity removal system, Top-20 Depletion ProteoPrep spin columns (Sigma), according to the manufacturer’s instructions and protocol. Protein depletion is carried out in untargeted proteomic analysis to identify low abundance proteins. Although commonly used, a limitation of protein depletionis that it cannot be expected to efficiently discover low abundance proteins because of non-linear depletion of albumin and can hinder downstream analysis.

### Protein precipitation

The depleted plasma proteins were mixed with ice-cold acetone/TCA (10% w/v) at a ratio of 1:4 and vortexed for 15 s. TCA/acetone precipitation was performed to remove interfering compounds and minimize plasma protein degradation. Protein precipitation was achieved after overnight incubation at − 20 °C. The mixture was then centrifuged at 12,000× g for 15 min at 4 °C.

### CyDye labelling, two-dimensional (2D) electrophoresis and image scanning

The protein pellets were each resuspended in labelling buffer (7 M urea, 2 M thiourea, 4% CHAPS, 30 mM Tris), and the pH was adjusted to 8.5. Protein concentrations were determined in triplicate using a 2D-Quant Kit (GE Healthcare, Sweden). The proteins were labelled with CyDye™ DIGE Fluor minimal dye (400 pmol/50 μg) (GE Healthcare, Sweden) according to the manufacturer’s recommendations, as previously described by our group [27–30]. The labelled samples were combined according to the experimental design (Table S2; Additional File 1). 2D-DIGE followed by first-dimensional analytical gel electrophoresis was performed as described previously [27–30]. Preparative gels were prepared using total protein (1 mg) obtained from a pool of equal protein amounts from the 34 plasma samples (10 EC, 12 HY, and 12 CO). The gels were stained for five days and then briefly rinsed with Milli-Q water before being stored until the spots could be identified using MS, as described previously [27–30].

### Statistical analysis

Automated spot detection was used to analyse 2D-DIGE gel images using Progenesis SameSpots software (Nonlinear Dynamics, UK). The software incorporated modules for gel warping, DIGE normalization, and comparison. To safeguard against data loss, all gel images were aligned and overlaid with a reference gel. The software determined the normalized volume (NV) of each spot on each gel based on the Cy3 (or Cy5) to Cy2 spot volume ratio. To ensure data normality, a logarithmic transformation was applied to the spot volumes, resulting in log-normalized volume (LNV), which served as the basis for quantifying differential expression. Direct comparisons were made between the EC, Hy, and CO groups, with fold difference values and *p* values determined using one-way analysis of variance (ANOVA). Prior to applying the statistical criteria (ANOVA, *p* ≤ 0.05, fold change ≥ 1.5), all the spots were subjected to prefiltering and manual inspection. Statistical processing employed normalized spot volumes rather than spot intensities. Only spots meeting the aforementioned statistical criteria were subjected to mass spectrometry (MS) analysis. The MS proteomics data for the study were processed using MetaboAnalyst version 5.0. The raw data were normalized to the median of the total sample, log-transformed, and Pareto-scaled to ensure that all signals were Gaussian distributed. Univariate analysis using volcano plot analysis was conducted for each binary comparison to identify significantly differentially expressed proteins based on a fold change criterion of greater than 1.5 or less than 0.67 and an FDR-adjusted *p* value of less than 0.05. The x-axis of the volcano plot represents the fold change (FC) between the groups, while the y-axis represents the *p* value. Multivariate analysis (orthogonal partial least squares discriminant analysis (OPLS-DA)) was performed to identify any clustering or separation between the compared datasets. The potential biomarkers were assessed for their sensitivity and specificity using receiver operating characteristic (ROC) curves based on the OPLS-DA method (MetaboAnalyst software version 5.0).

### Protein identification using MALDI-TOF mass spectrometry

Coomassie-stained gel spots were excised manually, washed, and digested according to previously described methods [27–30]. A mixture of tryptic peptides (0.8 μL) derived from each protein was spotted onto a MALDI target (384 MTP Anchorchip; 800 μm An-chorchip; Bruker Daltonics, Bremen, Germany). MALDI-MS spectra were obtained using an UltraflexTOF mass spectrometer equipped with a LIFT-MS device (Bruker Daltonics) at reflector and detector voltages of 21 kV and 17 kV, respectively, as described previously [27–30]. Peptide mass fingerprints (PMFs) were calibrated against standards and were searched using the Mascot search algorithm (v2.0.04, updated on 09/05/2023; Matrix Science Ltd., UK). The identified proteins were considered correct if they had a Mascot score greater than 56. The Mascot significance score was calculated using the formula Protein score = -10*Log(P), where P is the probability that the observed match is a random event; a protein score greater than 56 was considered significant (*p* ≤ 0.05). ID proteins with low scores were excluded because they were mostly random matches and were not significant (*p* > 0.05). Not all spots of interest could be identified because the proteins were present at such low concentrations, did not generate sufficiently intense mass fingerprints for analysis, or multiple proteins were present in the same spot, complicating the identification process [27–30].

### Bioinformatics analysis

The STRING database (https://string-db.org/) was used to construct a protein‒protein interaction (PPI) network of differentially expressed proteins, facilitating the analysis of protein interaction networks and the functions of differentially expressed plasma proteins in EC, Hy, and CO samples. The STRING database integrates UniProt IDs into the Ingenuity knowledge base, the largest manually curated resource that consolidates information from various published scientific studies. This software aids in identifying functions and pathways that are closely associated with the MS-generated protein list by superimposing the experimental expression data onto networks derived from published interactions. In addition, the PANTHER (protein analysis through evolutionary relationships) classification system (http://www.pantherdb.org) was used to categorize the identified proteins based on their molecular function and biological process.

## Results

### Proteomic analysis and identification of differentially expressed proteins

**Fig. 1:**
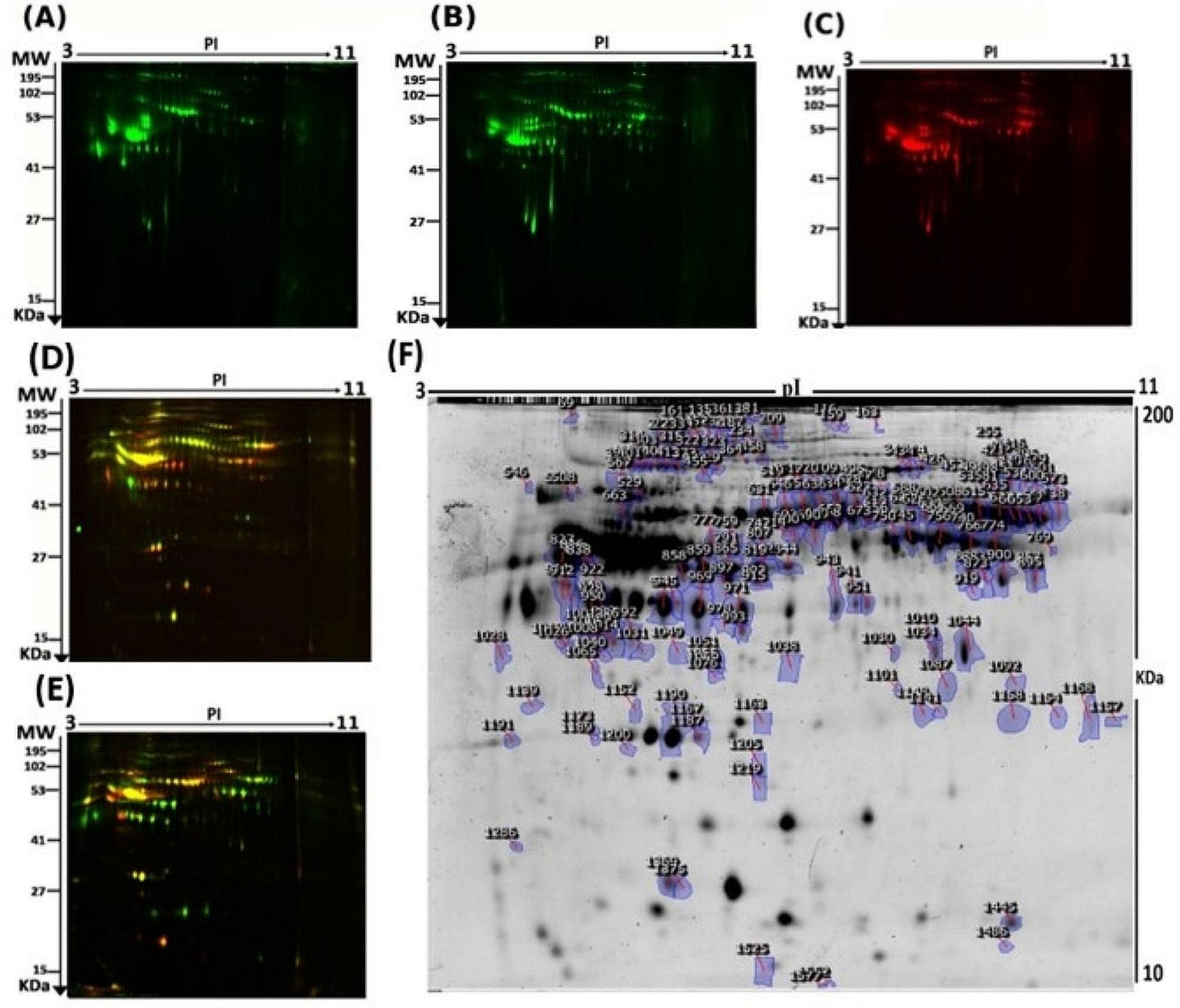
Representative fluorescent protein profiles of 2D-DIGE: (**A**) Control samples labelled with Cy3, (**B**) Hyperplasia samples labelled with Cy3, (**C**) Endometrial cancer samples labelled with Cy5, (**D**) Merged image of Hyperplasia/control samples labelled with Cy3/Cy5, (**E**) Endometrial cancer/control samples labelled with Cy3/Cy5, (**F**) Representative image of protein spots between different study groups. Numbered spots indicate those that were significantly differentially expressed between groups (≥ 1.5-fold change, *p* < 0.05)

To assess the differential protein expression among the EC, Hy and CO groups (34 samples from 17 gels), we performed 2D-DIGE and MALDI-TOF MS. Proteins were separated on IPG strips (pH 3–11) in the first dimension, followed by 12.5% PAGE in the second dimension. The gels were scanned with a Sapphire Biomolecular Imager (Azure Biosystems, Dublin, OH, USA) and digitalized via the image analysis software Sapphire Capture system (Azure Biosystems, Dublin, OH, USA). Figure 1 shows the representative fluorescent protein profiles of a 2D-DIGE of CO samples labelled with Cy3 (Fig. 1A), Hy samples labelled with Cy3 (Fig. 1B), and an EC labelled with Cy5 (Fig. 1C) The merged 2D-DIGE gel image of Hy/CO samples labelled with Cy3/Cy5 (Fig. 1D) and merged 2D-DIGE gel image of EC/CO samples labelled with Cy3/Cy5 (Fig. 1E) were then constructed.

The gels revealed a total of 1,151 spots, among which 173 were significantly different (ANOVA, *P* ≤ 0.05; fold change ≥ 1.5) between the CO, Hy and EC groups (Fig. 1F). The spot patterns were reproducible across all 17 gels, leading to alignment and further analysis. Normalization across the complete set of gels and quantitative differential analysis of the protein levels were achieved using an internal standard with Cy2 labelling. The spots showing statistical significance between the study groups were then manually excised from the preparative gel for protein identification by MS.

Peptide mass fingerprints (PMFs) successfully identified 114 out of the 173 protein spots excised from the preparative gel. MALDI-TOF mass spectrometry identified 42 spots as unique protein sequences that were matched to entries in the SWISS-PROT database by Mascot with high confidence scores. The sequence coverage of the proteins identified by PMF ranged from 8 to 69% (Table S3, Table S4; Additional File 1). In a few cases, variants of the same protein were found at several locations on the gel (Table S3; Additional File 1, Fig. 1F). A total of 85 differentially expressed proteins were identified between EC and CO. Among the differentially expressed proteins, 39 proteins were upregulated, and 46 were downregulated. The significantly upregulated proteins included vitamin D-binding protein, haptoglobin, ceruloplasmin, alpha-2-macroglobulin, and kinogen-1. The significantly downregulated proteins included complement C3, alpha-1-antitrypsin, alpha-1B-glycoprotein, and zinc-alpha-2-glycoprotein, among others. Among the identified proteins, 17 proteins (ceruloplasmin, antithrombin-III, alpha-1-antitrypsin, kinogen-1, apolipoprotein A-I, vitamin D-binding protein, complement C3, etc.) were found in more than one spot on the gels, which could be associated with their posttranslational modifications, cleavage by enzymes, or the presence of different protein species (Table S4A; Additional File 1). A total of 81 differentially expressed proteins were identified in Hy vs. CO. Among the differentially expressed proteins, 40 proteins were upregulated, and 41 were downregulated. The significantly upregulated proteins included vitamin D-binding protein, serotransferrin, plasminogen, kininogen-1, and haptoglobin, among others. The significantly downregulated proteins included alpha-1-antitrypsin, alpha-1B-glycoprotein, apolipoprotein A-I, ceruloplasmin, and complement C3, among others. Among the identified proteins, 18 proteins (ceruloplasmin, haptoglobin, antithrombin-III, alpha-1-antitrypsin, alpha-1-antichymotrypsin, kinogen-1, etc.) were found in more than one spot on the gels, which could be associated with their posttranslational modifications, cleavage by enzymes, or the presence of different protein species (Table S4B, Additional File 1). A total of 33 differentially expressed proteins identified in EC vs. Hy. Among the differentially expressed proteins, 12 were upregulated, and 21 were downregulated. The significantly upregulated proteins included haptoglobin, apolipoprotein A-I, apolipoprotein A-IV, transthyretin, and zinc-alpha-2-glycoprotein, among others. The significantly downregulated proteins included ceruloplasmin, kinogen-1, and haemopexin, alpha-1-antichymotrypsin, among others. Among the identified proteins, 7 proteins (Haptoglobin, alpha-1-antichymotrypsin, kinogen-1, serotransferrin, haemopexin, etc.) were found in more than one spot on the gels, which could be associated with their posttranslational modifications, cleavage by enzymes, or the presence of different protein species (Table S4C; Additional File 1).

### Overall proteomic analysis between study groups

**Fig. 2:**
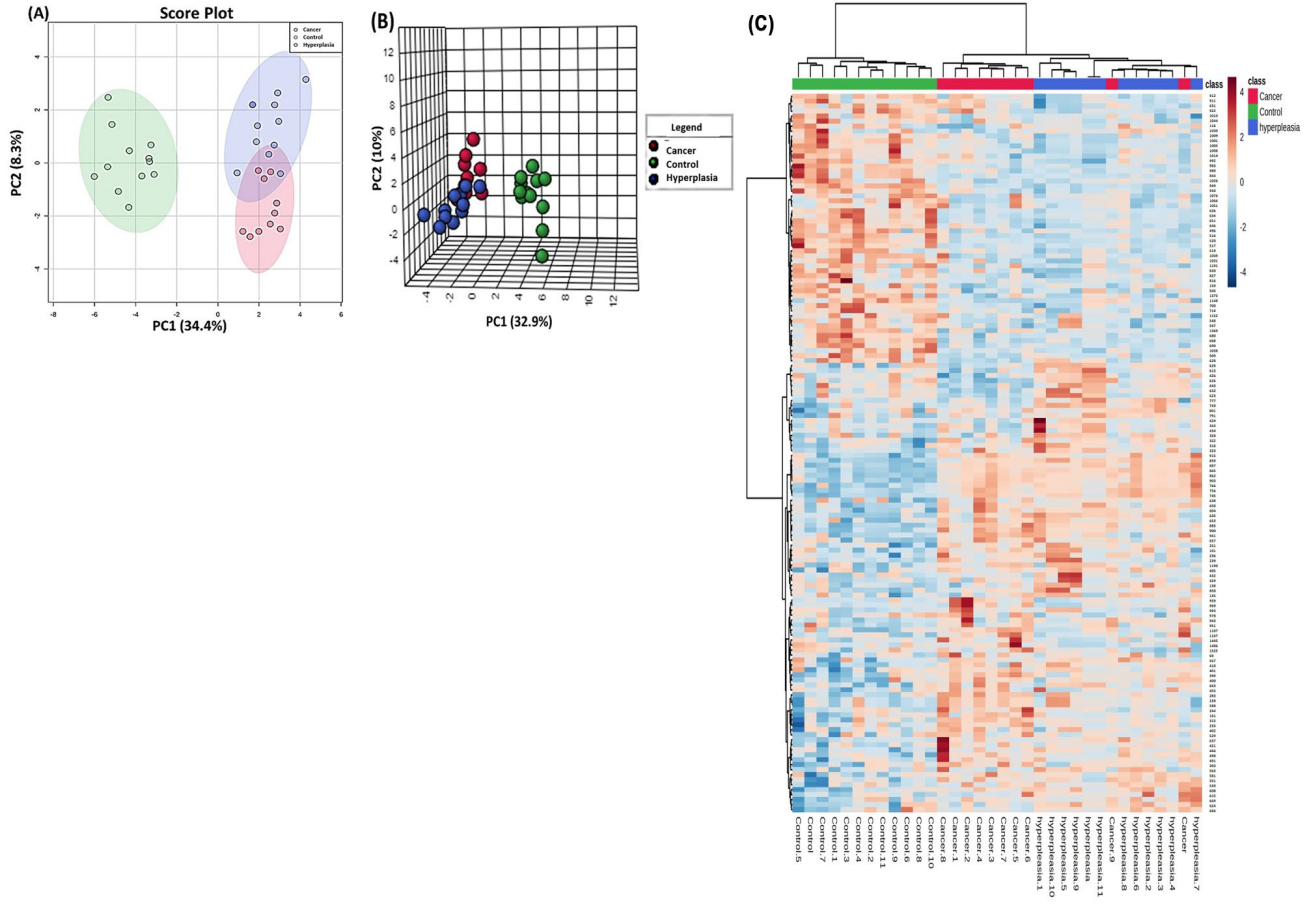
Proteomic profiling of control (CO), hyperplastic (Hy), and endometrial cancer (EC) patients. (**A**) Two − dimensional principal component analysis (2D PCA) score plots of significantly differentially expressed plasma proteins identified between the three groups of the study. The CO, Hy, and EC samples are represented as green, blue, and red circles, respectively. (**B**) Three − dimensional principal component analysis (3D PCA) score plots of significantly differentially expressed plasma proteins identified between the three groups of the study. The CO, Hy, and EC samples are represented as green, blue, and red circles, respectively. (**C**) Heatmap analysis of identified proteins that were significantly altered between the CO (green), Hy (blue) and EC (red) groups. The colour range bar indicates downregulated proteins (blue) and upregulated proteins (brown)

The score plots obtained for all three study groups are shown in Fig. 2A. Two − dimensional principal component analysis (2D PCA) of the significantly differentially expressed plasma proteins identified was used to visualize each study group and detect outliers. The primary source of variance (PC1, 34.4%, and PC2, 8.3%) allows separation of the EC (pink circles), Hy (blue circles) and CO (green circles). Three − dimensional principal component analysis (3D PCA) score plots of significantly differentially expressed plasma proteins identified between the three groups of the study are shown in Fig. 2B. The heatmap clearly revealed proteins with significant differences between the CO + EC group and the CO + Hy group (Fig. 2C). Therefore, these genes could be candidate biomarkers for the identification of Hy and EC.

### Proteomic profiling of EC and CO

**Fig. 3:**
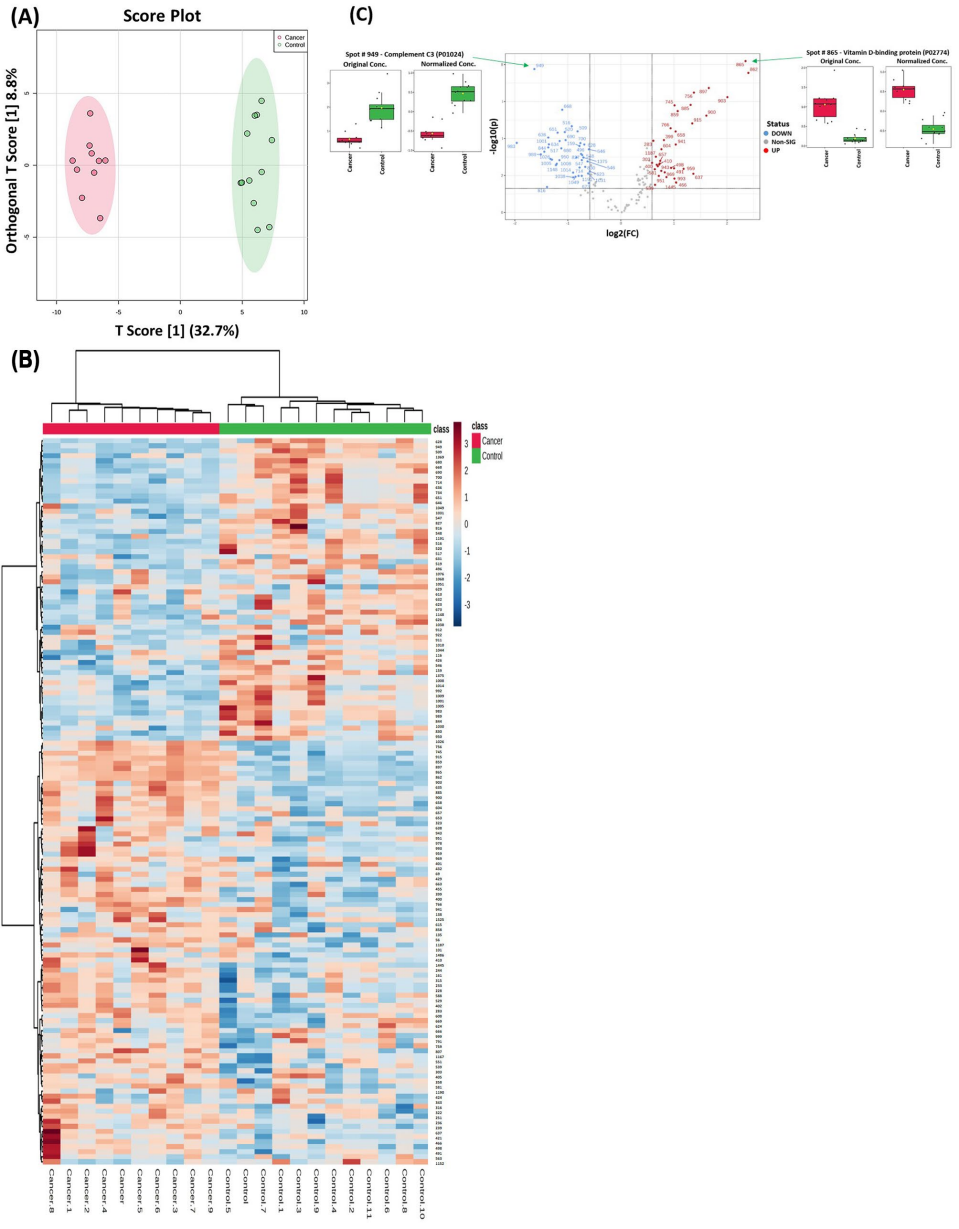
Proteomic profiling of endometrial cancer (EC) and control (CO) patients. (**A**) The orthogonal partial least squares-discriminant analysis (OPLS-DA) score plot showed evident separation between the two groups (EC and CO). The robustness of the created models was evaluated by the fitness of the model R2Y = 0.987 and predictive ability (Q2 = 0.922) values. The EC and CO samples are represented as red and green circles, respectively. (**B**) Heatmap analysis of identified proteins that were significantly altered between the control (green) and EC (red) groups. The colour range bar indicates downregulated proteins (blue) and upregulated proteins (brown). between the EC and CO groups. (**C**) The volcano plot shows a significant change in the levels of several proteins, of which red represents upregulated and blue represents downregulated plasma proteins in the EC group compared with the control group (FDR *p* value ≤ 0.05, fold change ≥ 1.5). Box-whisker plots show one of the identified downregulated proteins (Spot # 949 - Complement C3 (P01024)) and one of the upregulated proteins (Spot # 865 - Vitamin D-binding protein (P02774)) (FDR *p* ≤ 0.05 and fold change ≥ 1.5) in EC in comparison with CO

The protein biomarkers identified between EC and CO are shown in Fig. 3. A supervised, multivariate technique, OPLS-DA, was used. A score plot of the OPLS-DA model is shown in Fig. 3A. The EC group was clearly separated from CO, indicating the potential efficiency of plasma proteomics for identifying EC. Cross-validated R2Y and Q2 coefficients between EC and CO were calculated. The robustness of the created models was evaluated by the fitness of the model (R2Y = 0.987) and the predictive ability (Q2 = 0.922) (Figure S1). Box whisker plots show one of the downregulated proteins (Spot # 949 - Complement C3 (P01024)) and one of the upregulated proteins (Spot # 865 - Vitamin D-binding protein (P02774)) in the EC group compared with the CO group (Fig. 3C).

### Proteomic profiling between Hy and CO

**Fig. 4:**
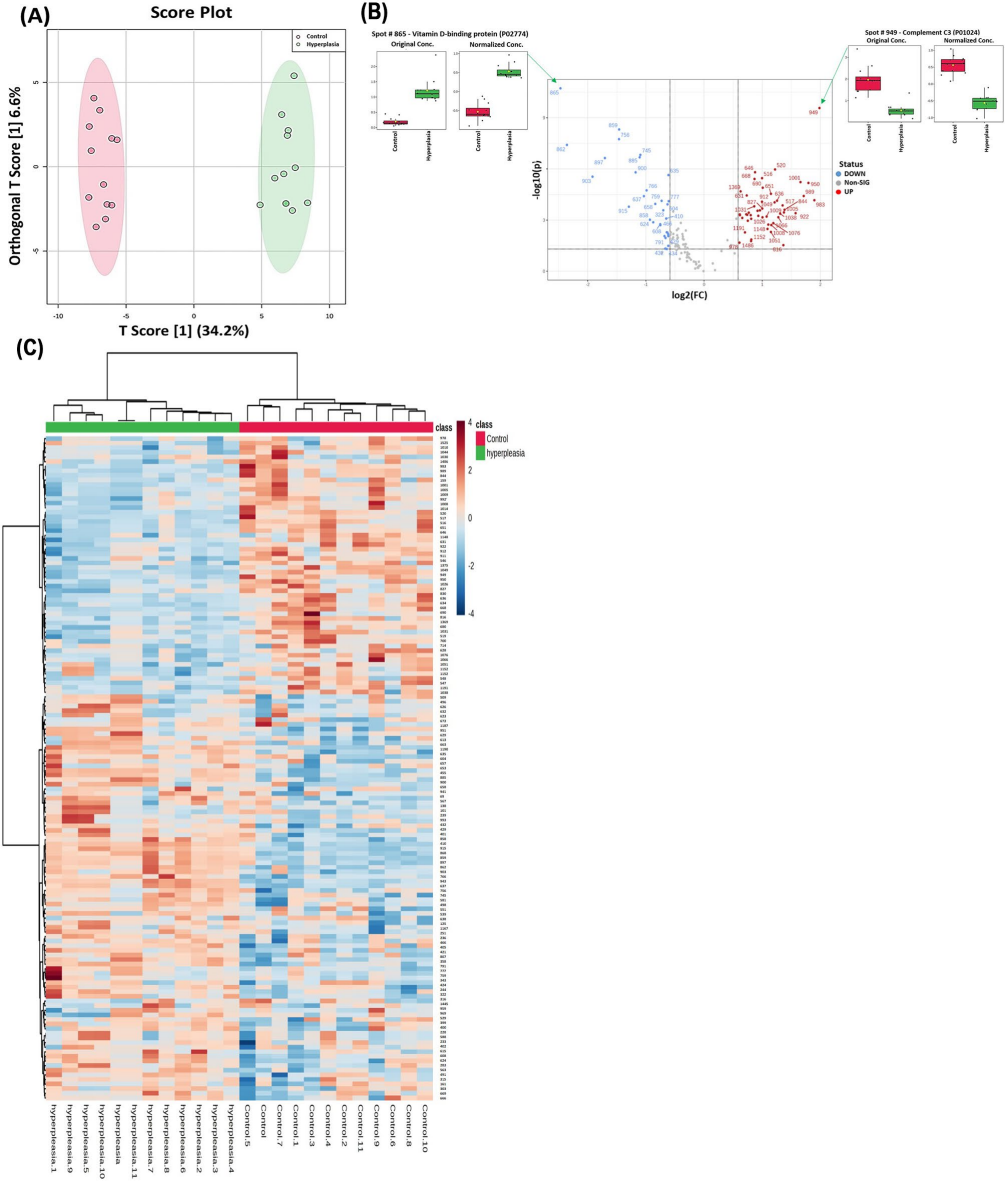
Proteomic profiling of the Hyperplasia (Hy) and Control (CO) groups. (**A**) Orthogonal partial least squares-discriminant analysis (OPLS-DA) score plot showing evident separation between the Hy and CO groups. The robustness of the created models was evaluated by the fitness of the model R2Y = 0.988 and predictive ability (Q2 = 0.945) values. The Hy and CO samples are represented as red and green circles, respectively. (**B**) Heatmap analysis of identified proteins that were significantly altered between the control (red) and Hy (green) groups. The colour range bar indicates downregulated proteins (blue) and upregulated proteins (brown). (**C**) The volcano plot shows a significant change in the levels of several proteins, of which red represents upregulated and blue represents downregulated plasma proteins in the Hy and CO groups (FDR *p* value ≤ 0.05, fold change ≥ 1.5). Box-whisker plots show one of the identified downregulated proteins (Spot # 949 - Complement C3 (P01024)) and one of the upregulated proteins (Spot # 865 - Vitamin D-binding protein (P02774)) (FDR *p* ≤ 0.05 and fold change ≥ 1.5) in the Hy group compared with the CO group

The protein biomarkers that differentiated between Hy and CO samples are shown in Fig. 4. A supervised, multivariate technique, OPLS-DA, was used. A score plot of the OPLS-DA model is shown in Fig. 4A. The Hy group was clearly separated from the CO group, demonstrating the effectiveness of plasma proteomics in distinguishing Hy from CO samples. The robustness of the generated models was assessed by the high R2Y (0.988) and Q2 (0.945) values, indicating that the models could accurately predict the separation between Hy and CO samples (Figure S1). Box whisker plots show one of the identified downregulated proteins (Spot # 949 - Complement C3 (P01024)) and one of the upregulated proteins (Spot # 865 - Vitamin D-binding protein (P02774)) (FDR *p* ≤ 0.05 and fold change ≥ 1.5) in the Hy group compared with the CO group (Fig. 4C).

### Proteomic profiling between EC and Hy

**Fig. 5:**
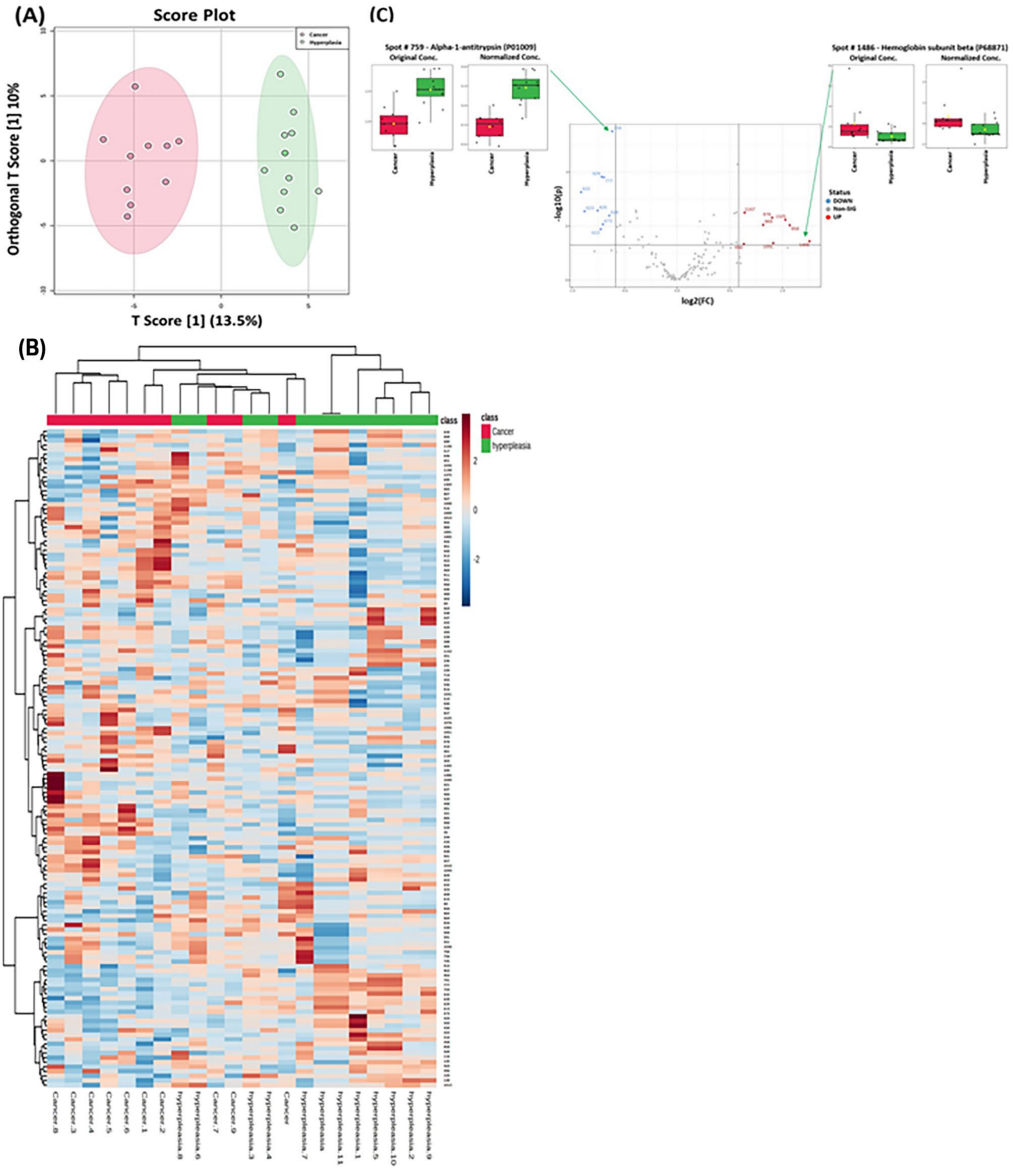
Proteomic profiling of endometrial cancer (EC) and hyperplasia (Hy) patients. (**A**) Orthogonal partial least squares-discriminant analysis (OPLS-DA) score plot showing evident separation between the two groups of patients with EC and Hy. The robustness of the created models was evaluated by the fitness of the model R2Y = 0.947 and the predictive ability (Q2 = 0.706) values. The EC and Hy samples are represented as red and green circles, respectively. (**B**) Hierarchical clustering (HAC) and heatmap analysis of identified proteins that were significantly altered between the EC (red) and Hy (green) groups. The colour range bar indicates downregulated proteins (blue) and upregulated proteins (brown). (**C**) The volcano plot shows a significant change in the levels of several proteins, of which red represents upregulated and blue represents downregulated plasma proteins in the EC and Hy groups (FDR *p* value ≤ 0.05-fold change ≥ 1.5). Box-whisker plots show one of the identified downregulated proteins (Spot # 759 - Alpha-1-antitrypsin (P01009)) and one of the upregulated proteins (Spot # 1486 - Haemoglobin subunit beta (P68871)) (FDR *p* ≤ 0.05 and fold change ≥ 1.5) in EC

The potential biomarkers that differed between EC and Hy are shown in Fig. 5. A supervised, multivariate technique, OPLS-DA, was used. A score plot of the OPLS-DA model is shown in Fig. 5A. A few proteins present in the EC group were clearly separated from Hy, indicating the potential efficiency of plasma proteomics for distinguishing EC from Hy. Cross-validated R2Y and Q2 values between EC and Hy were calculated. The robustness of the created models was evaluated by the fitness of the model (R2Y = 0.947) and the predictive ability (Q2 = 0.706) (Figure S1C; Additional File 1). Heatmap analysis revealed proteins whose expression significantly differed between the Hy (green) and EC (red) groups (Fig. 5B). The heatmap clearly shows the proteins with a significant difference between the EC and Hy groups. Consequently, these proteins show promise as candidate biomarkers for identifying EC. A volcano plot analysis was performed to compare the EC and Hy groups, employing a moderate t test (*p* value < 0.05) and a fold change cut-off of 1.5. This analysis revealed that 12 proteins were upregulated and 21 proteins were downregulated in the EC group compared to the Hy group (Fig. 5C). Box-whisker plots illustrate one of the identified downregulated proteins (Spot #759 - Alpha-1-antitrypsin (P01009)) and one of the identified upregulated proteins (Spot #1486 - Haemoglobin subunit beta (P68871)) (FDR *p* ≤ 0.05 and fold change ≥ 1.5) in the EC group compared to the Hy group (Fig. 5C).

### Evaluation of protein biomarkers between study groups

**Fig. 6:**
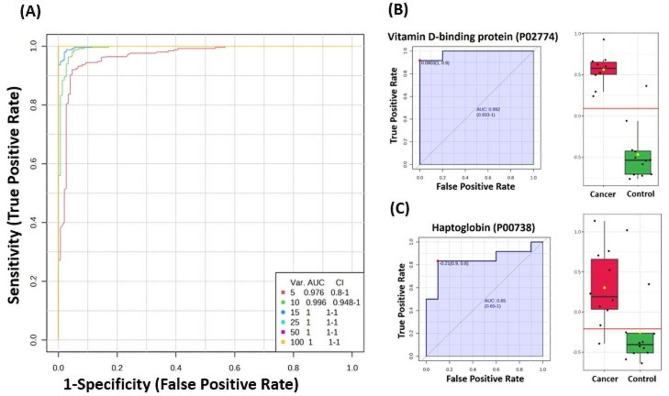
Results of biomarker evaluation in endometrial cancer (EC) patients and controls (COs). (**A**) The receiver operating characteristic (ROC) curve was generated by the OPLS-DA model, with area under the curve (AUC) values calculated from the combination of 5, 10, 15, 25, 50, and 100 proteins. (B, C) Two upregulated proteins in EC with the highest AUC: (**B**) Vitamin D-binding protein, AUC = 0.992; Box plot (FDR *p* ≤ 0.05 and fold change ≥ 1.5), where red represents EC and green represents control (**C**) haptoglobin, AUC = 0.85; Box plot (FDR *p* ≤ 0.05 and fold change ≥ 1.5), where red represents EC and green represents control

The selected frequency plots representing the 10 significant proteins with the highest variable influence on projection (VIP) scores in the OPLS-DA model according to their level in different group comparisons are shown in Figure S2; Additional File 1. A frequency plot depicting the abundance of 10 identified proteins in the EC and CO samples revealed the presence of proteins, including vitamin D-binding protein, alpha-1-antitrypsin, haptoglobin, and complement C3. (Figure S2A; Additional File 1). Multivariate exploratory ROC analysis based on the identified common and significantly dysregulated proteins between the EC and CO groups was performed using OPLS-DA as a classification and feature ranking method. The AUC of the exploratory ROC curve for the top ten variants (proteins) was 0.996 (Fig. 6A). The AUCs of two proteins from the top ten variants (proteins): vitamin D-binding protein (P02774) (AUC of 0.992). Box-whisker plots show an FDR *p* ≤ 0.05 and a fold change ≥ 1.5, where red represents EC and green represents CO (Fig. 6B) and haptoglobin (P00738) (AUC of 0.85). Box whisker plots show FDR *p* ≤ 0.05 and fold change ≥ 1.5, where red represents EC and green represents CO (Fig. 6C).

A frequency plot (Figure S2B; Additional File 1) illustrates the abundance of 10 identified proteins in the Hy and CO samples, highlighting several notable proteins, including vitamin D-binding protein, serotransferin, alpha-1-antichymotrypsin, and complement C3. Additionally, a multivariate exploratory ROC analysis was conducted using OPLS-DA as a classification and feature ranking method, focusing on the commonly identified and significantly dysregulated proteins between the Hy and CO groups. The AUC of the exploratory ROC curve for the top ten variants (proteins) was 1.00 (Figure S3A; Additional File 1). The AUCs of two proteins from the top ten variants (proteins): vitamin D-binding protein (P02774) (AUC of 1.00), for which the box-whisker plots show an FDR *p* ≤ 0.05 and fold change ≥ 1.5, where red represents CO and green represents Hy (Figure S3B; Additional File 1) and serotransferrin (P02787) (AUC of 1.00), for which the box-whisker plots show an FDR *p* ≤ 0.05 and fold change ≥ 1.5, where red represents CO and green represents Hy (Figure S3C; Additional File 1).

The top 10 proteins identified according to their levels in EC versus Hy, including apolipoprotein A-1, alpha-1-antichymotrypsin, ceruloplasmin, and alpha-1-antitrypsin, are represented in the frequency plot (Figure S2C; Additional File 1). Multivariate exploratory ROC analysis based on the identified common and significantly dysregulated proteins between the EC and Hy groups was performed using OPLS-DA as a classification and feature ranking method. Among the top ten protein variants, two exhibited particularly high AUC values: alpha-1-antichymotrypsin (P01011), with an AUC of 0.95, and apolipoprotein A-1 (P02647), with an AUC of 0.908. Box whisker plots (Figure S4B and S4C; Additional File 1) depict the expression levels of these proteins in EC and Hy samples, with red representing EC and green representing Hy. The proteins were identified with FDR *p* ≤ 0.05 and fold change ≥ 1.5.

### Protein–protein interaction networks

**Fig. 7:**
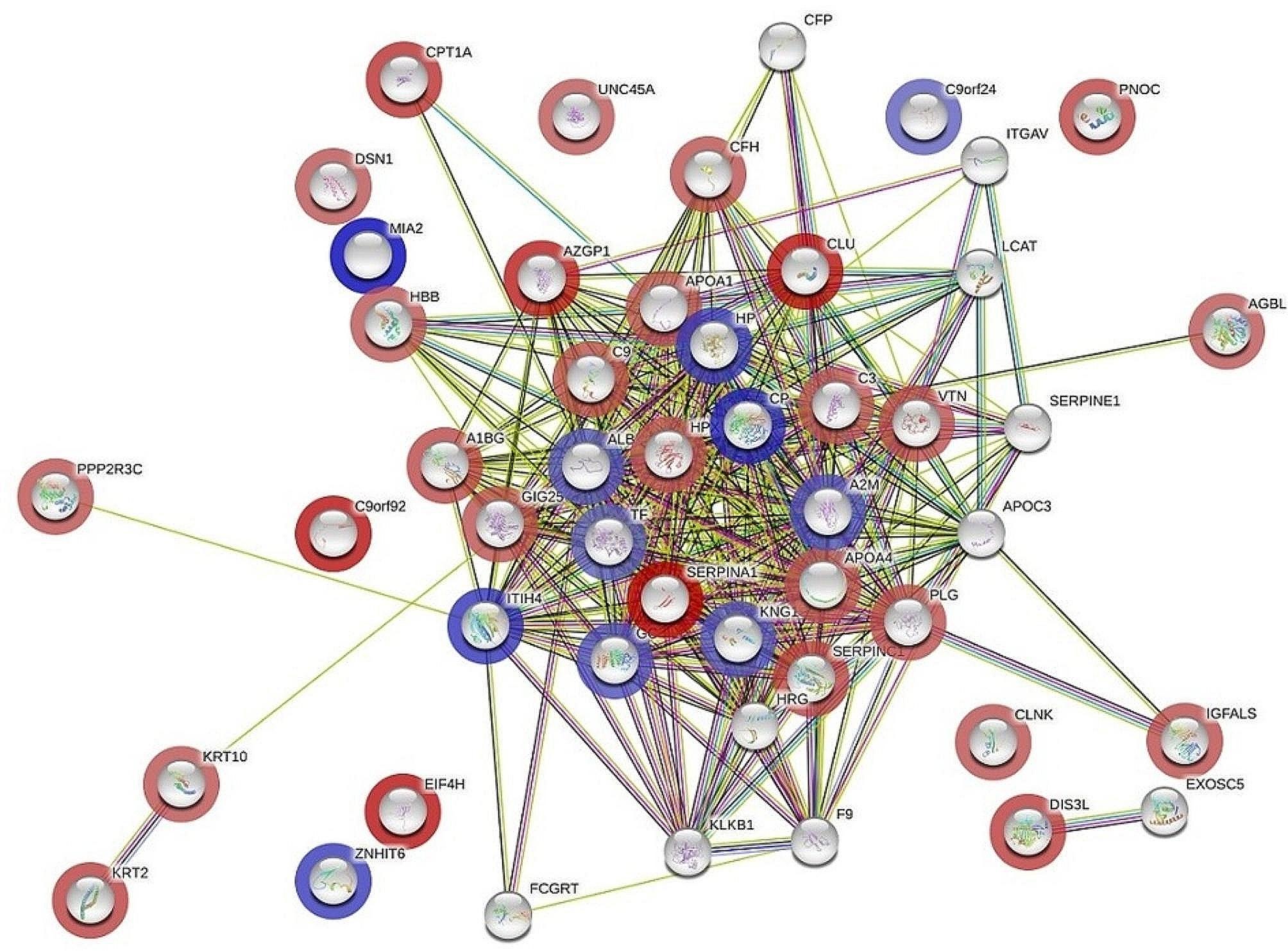
The most enriched interaction network of the differentially expressed proteins in the control and endometrial cancer groups. The protein nodes with blue halos indicate upregulated genes; the protein nodes with red halos indicate downregulated genes. Protein nodes without a halo were identified by the STRING database, which indicated potential targets that were functionally coordinated with the differentially expressed proteins. The solid black line indicates coexpression; the green line indicates the gene neighbourhood; the dark blue line indicates gene co-occurrence; and the purple line indicates experimentally determined protein interactions. The central nodes of pathways related to signalling of complement and coagulation cascades, regulation of insulin-like growth factor (IGF) transport and uptake by insulin-like growth factor binding proteins (IGFBPs), and plasma lipoprotein assembly, remodelling, and clearance (KEGG, Reactome databases) were found to be dysregulated between the two states

The protein‒protein interactions among the significantly differentially abundant proteins in the EC, Hy, and CO groups were analysed using the STRING database (Fig. 7and Figure S5A and B; Additional File 1). STRING database analysis revealed distinct canonical pathways for each protein interaction network, as summarized in Table S5; Additional File 1. The identified proteins exhibited significant interactions with each other, forming interconnected networks. Notably, the pathways enriched in the EC vs. CO comparison included signalling of complement and coagulation cascades (FDR 5.98E-13), regulation of insulin-like growth factor (IGF) transport and uptake by IGF binding proteins (FDR 6.16E-11), and plasma lipoprotein assembly, remodelling, and clearance (FDR 0.0025). Similar pathway enrichments were observed in the Hy vs. CO and EC vs. Hy comparisons, albeit with varying statistical significance (Table S5 A, B, C; Additional File 1).

### Protein analysis through an evolutionary relationship

**Fig. 8:**
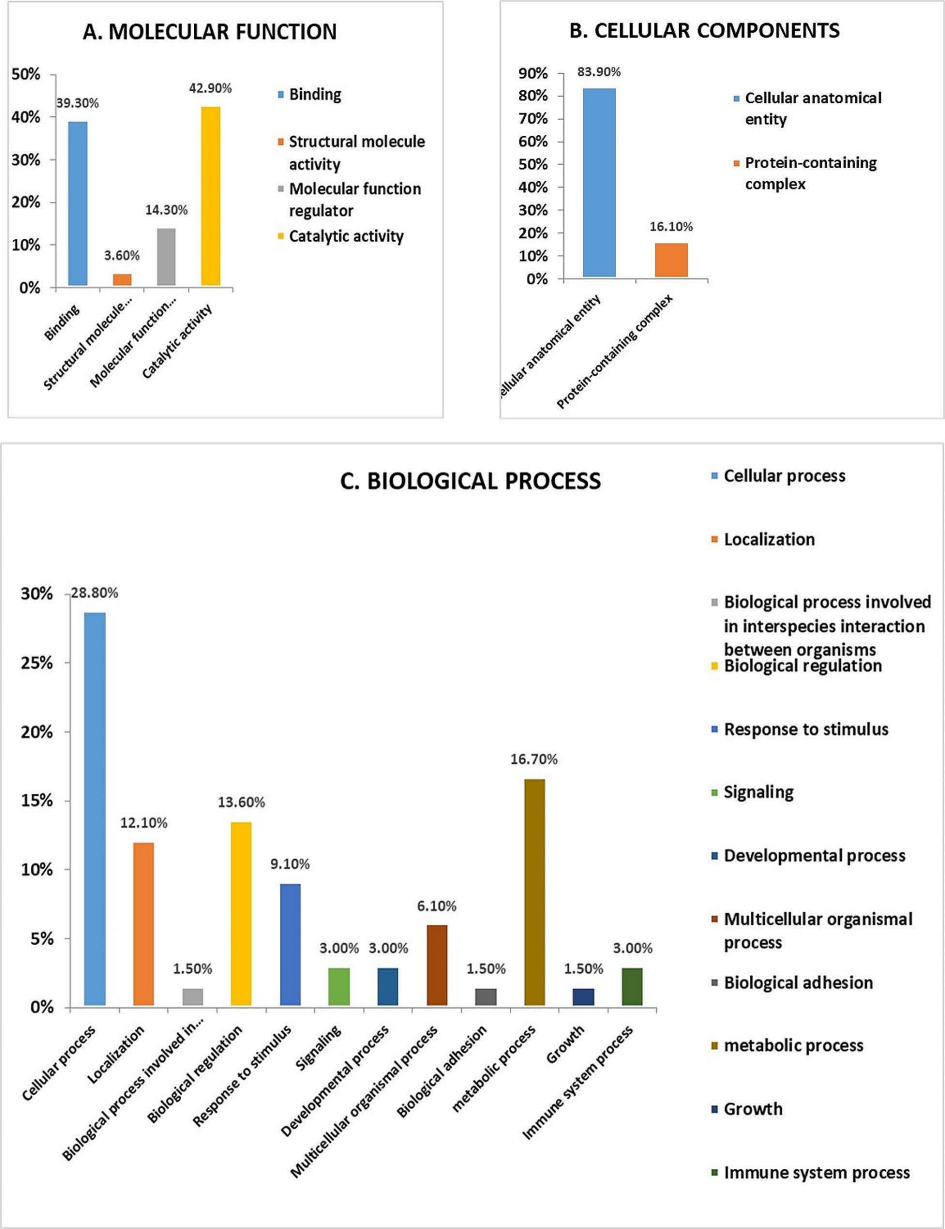
Comparative depiction (%) of identified proteins categorized into groups according to their molecular function (**A**), cellular component (**B**), and biological process (**C**)

The PANTHER (protein analysis through evolutionary relationships) classification system was used to categorize the identified proteins based on their molecular function (Fig. 8A), cellular localization (Fig. 8B), and biological process (Fig. 8C). The functional classification revealed that enzymes with catalytic activity (42.9%) and proteins with binding activity (39.3%) constituted the majority of the differentially expressed proteins (Fig. 8A). A significant portion of the identified proteins were localized within cellular anatomical entities (83.9%) (Fig. 8B). In terms of biological process, the identified proteins were primarily involved in cellular processes (28.8%), followed by metabolic processes (16.7%) (Fig. 8C).

## Discussion

This study represents an untargeted proteomic approach using 2D-DIGE coupled with MALDI-TOF mass spectrometry and bioinformatics analysis to investigate the proteomic profiles of the EC, Hy, and CO groups utilizing plasma samples from Saudi women. Previous studies have identified limited biomarkers of EC in plasma, serum, and tissue samples but have failed to establish the functional role of these proteins in EC development. Even fewer of these proteins have been linked to EC and its progression, and none have been successfully translated into clinical applications [31, 32]. Enhancing our understanding of the underlying molecular mechanisms is crucial for discovering more reliable diagnostic and prognostic biomarkers for EC. It is therefore crucial to expand on the molecular pathology underlying this disease to identify more informative diagnostic and predictive markers for EC. In our present study, we identified 114 significantly abundant proteins, with 85 proteins (39 upregulated and 46 downregulated) showing differential expression in EC patients, while 81 proteins (40 upregulated and 41 downregulated) exhibited differential expression in Hy patients compared to CO patients. Additionally, we identified 33 proteins (12 upregulated and 21 downregulated) that were significantly more dysregulated in Hy patients than in EC patients.

### Evaluation of proteomic biomarkers for discriminating between ECs and CO patients

Our study revealed a more than 3-fold increase in vitamin D binding protein in ECs compared with COs. Vitamin D binding protein is a multifunctional protein that plays a vital role in the transport and metabolism of vitamin D and helps transport and store other hormones and nutrients, and plays a role in immune function, cell growth, and protection against cell damage [33]. It can activate macrophages and increase the movement of monocytes and neutrophils. Several studies have indicated that elevated levels of vitamin D binding protein are present in many malignant diseases, including colorectal cancer [34], lung cancer [34], bladder cancer [35], pancreatic cancer [36], and ovarian cancer [37]. Although the relationship between VDBP and EC is not fully understood, further.

The expression of another protein, complement C3, was significantly downregulated in ECs compared with that in COs. The complement system is a complex network of proteins involved in innate immunity that help the body fight cancer by recruiting white blood cells to the tumour, killing tumour cells, releasing proinflammatory mediators [38]. Activation of the complement protein C3 in human gastric cancer (GC) is characterized by the localized buildup of C3 and its effectors, as well as a decrease in plasma C3 levels, which appears to contribute to tumour progression and poor prognosis in patients with GC [39]. C3 can also activate the JAK2/STAT3 signalling pathway, that is hypothesized to lead to poorer cancer outcomes [40].

Our study showed that Hp was upregulated in EC by more than 3-fold, in contrast to CO. Haptoglobin (Hp) is an acute reactant protein [41], and scavenges haemoglobin during intravascular or extravascular haemolysis events [42]. Hp is an important antioxidant and anti-inflammatory proteins whose elevated levels have been noted in many malignant diseases, including breast cancer [43], and EC [44]. Melanoma inhibitory activity (MIA) was upregulated by approximately 3.45-fold in the EC group compared with the CO group. MIA is a secretory protein that promotes cell separation, migration, invasion, and metastasis and inactivates cancer cell apoptosis [45, 46]. It is expressed in several cancers, including pancreatic cancer [47], and breast cancer [48], suggesting that MIA2 may be involved in EC carcinogenesis. VDBP, C3, Hp, A1BG, and MIA2 could be used as prognostic biomarkers for detecting EC, as shown by canonical pathway analysis.

### Evaluation of proteomic biomarkers for discriminating between Hy and CO

The comparison between Hy and CO also revealed an increase in VDBP levels and a decrease in C3, similar to what was observed in EC. Interestingly, other proteins, including haemopexin and kininogen, were also significantly altered in this group.

Haemopexin (Hx) protein levels were downregulated compared with those in Hy plasma patients in our study. Hx is a 60 kDa plasma glycoprotein expressed primarily by the liver whose level increases due to stimulation by IL-6, IL-11, IL-1b, and tumour necrosis factor (TNF)-a [49]. A number of studies have demonstrated that Hx is overexpressed in different cancers, such as breast cancer [50], and ovarian cancer [51]. Hx is thought to be a tumour suppressor rather than a protein that promotes tumours. The role of Hx in cancer remains controversial.

The expression of Interalpha-trypsin inhibitor (ITIH) was upregulated in Hy patients in contrast to that in CO patients. The ITIH proteins originally isolated from human plasma are plasma serine protease inhibitor proteins involved in inflammation, tumorigenic processes, and metastasis [52]. Concentration of ITIH4 was significantly greater in the serum of breast cancer patients [53] and tissue of colorectal cancer patients [52]. Kininogen 1 (KNG1) was upregulated approximately 2.1-fold in Hy patients compared with CO patients. KNG1 is an inhibitor of cysteine proteins known to inhibit cell proliferation and angiogenesis and demonstrate a role in cancer development [54]. Previous studies have shown that KNG1 has antiangiogenic effects and inhibits endothelial cell proliferation [55]. Additionally, KNG1 was identified as a serum biomarker for the early detection of advanced CRC and oral squamous cell carcinoma [54, 56]. VDBP, C3, Hx, ITIH, and KNG1 could be used as prognostic biomarkers for EC patients for the detection of EC, as shown by canonical pathway analysis.

### Evaluation of proteomic biomarkers for discriminating between EC and Hy

A comparison between EC and Hy revealed a decrease in the level of α1-antitrypsin (A1AT) in the cancer group. A1-AT is a glycoprotein belonging to the family of serine protease inhibitors. Previous studies have indicated a significant increase in the levels of A1AT under malignant conditions [57], and a deficiency in this protein has been proposed to favour invasion by cancer cells. In our study, the levels of A1AT were greater in Hy than in EC, indicating that this protein is a good marker for separating these two states.

In our study, the protein level of serotransferrin and transthyretin (TTR), were increased in EC patients compared with Hy patients. Serotransferrin is a negative, acute-phase protein involved in physiological changes that cause various effects, including tissue damage, infections, and immune disorders, and reduces chronic inflammation and malignant growth [58]. Serotransferrin protein levels were significantly lower in patients ovarian and other gynaecological cancers [59, 60] and during inflammation [61], which is consistent with the findings of other previous studies. TTRs are proteins that bind to thyroid hormones and globules and transport thyroid hormones in the blood. Additionally, the serum TTR level has been reported to be a prognostic biomarker in pancreatic [62], and lung cancers [63]. A1AT, serotransferrin TTR, and a2M could be used as prognostic biomarkers for detecting EC, as shown by canonical pathway analysis.

Protein interaction analysis using STRING identified the top canonical pathways related to signalling of complement and coagulation cascades, regulation of (IGF) transport and uptake by (IGFBPs) and plasma lipoprotein assembly, remodelling, and clearance (Fig. 7). The involvement of these pathways differed among the three groups. The highest FDR for the involvement of proteins related to complement and coagulation cascades was for Hy vs. CO, followed by EC vs. Hy and finally EC vs. CO. On the other hand, the FDR for the regulation of insulin-like growth factor (IGF) transport and uptake by insulin-like growth factor binding proteins (IGFBPs) was highest for EC vs. Hy, followed by EC vs. CO, and then Hy vs. CO. The third most significant pathway, involving plasma lipoprotein assembly, remodelling, and clearance, was highest in EC vs. Hy, followed by Hy vs. CO and then EC vs. CO. The differences in the regulation of these pathways could provide insight into the progression of Hy to EC.

## Conclusion

The results of this study revealed significant differences in protein profiles between CO, Hy and EC plasma. Vitamin D binding protein and complement C3 distinguished Hy and EC from CO with the greatest changes in expression. The largest group of differentially expressed proteins identified was enzymes with catalytic activity. Protein interactions revealed three main biological processes: signalling of complement and coagulation cascades, regulation of insulin-like growth factor (IGF) transport and uptake by insulin-like growth factor binding proteins (IGFBPs), and plasma lipoprotein assembly, remodelling, and clearance. The identified plasma protein markers have the potential to serve as biomarkers for differentiating between EC and Hy, as well as for early diagnosis and monitoring of cancer progression. However, further studies evaluating these proteins are needed to determine whether these proteins could be potential biomarkers for use in the early diagnosis of patients with EC.

### Electronic supplementary material

Below is the link to the electronic supplementary material.

**Supplementary Material 1: Additional file 1: Figure S1**: Orthogonal partial least squares-discriminant analysis (OPLS-DA)-Permutation analysis; **Figure S2**: Frequency plot of 10 identified proteins; **Figure S3**: Biomarker evaluation in Hyperplasia and Controls; **Figure S4**: Biomarker evaluation in Endometrial cancer (EC) and Hyperplasia (Hy); Figure S5: The most enriched interaction network of the differentially expressed proteins in different groups; **Table S1**: Characteristics of study subjects; **Table S2**: Experimental design; **Table S3**: Mass spectrometry list of significant differentially abundant proteins; **Table S4**: Identified proteins, with changes in abundance of significantly differentially abundant proteins between cancer, hyperplasia and control states in plasma samples. **Table S5**: Different canonical pathways identified via STRING database analysis

## Data Availability

No datasets were generated or analysed during the current study.
