# Supplementary material for: Plasma-based proteomic profiling identifies the distinct regulation of proteins in hyperplasia and endometrial cancer
Source: BMC Cancer. 2024 Jun 20;24:752. doi: 10.1186/s12885-024-12522-0 (PMC11191338; doi:10.1186/s12885-024-12522-0)
Supplement: Supplementary file 1 — Supplementary Material 1: Additional file 1: Figure S1: Orthogonal partial least squares-discriminant analysis (OPLS-DA)-Permutation analysis; Figure S2: Frequency plot of 10 identified proteins; Figure S3: Biomarker evaluation in Hyperplasia and Controls; Figure S4: Biomarker evaluation in Endometrial cancer (EC) and Hyperplasia (Hy); Figure S5: The most enriched interaction network of the differentially expressed proteins in different groups; Table S1: Characteristics of study subjects; Table S2: Experimental design; Table S3: Mass spectrometry list of significant differentially abundant proteins; Table S4: Identified proteins, with changes in abundance of significantly differentially abundant proteins between cancer, hyperplasia and control states in plasma samples. Table S5: Different canonical pathways identified via STRING database analysis [file 12885_2024_12522_MOESM1_ESM.docx]

**Figure S1**: Orthogonal partial least squares-discriminant analysis (OPLS-DA)-Permutation analysis **(A)** An observed and cross-validated R2Y and Q2 coefficients between endometrial cancer and control. The robustness of the created models was evaluated by the fitness of the model R2Y = 0.987 and predictive ability Q2 = 0.922 values. **(B)**. An observed and cross-validated R2Y and Q2 coefficients between hyperplasia and control. The robustness of the created models was evaluated by the fitness of the model R2Y = 0.988 and predictive ability Q2 = 0.945 values. **(C)**. An observed and cross-validated R2Y and Q2 coefficients between endometrial cancer and hyperplasia. The robustness of the created models was evaluated by the fitness of the model R2Y = 0.947 and predictive ability Q2 = 0.706 values.

**
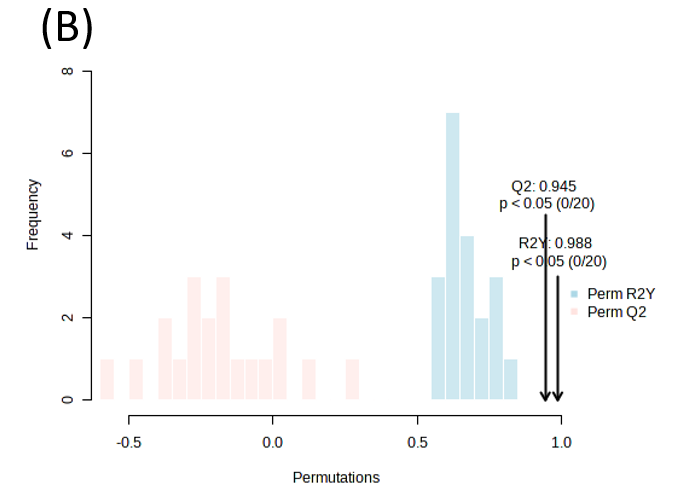

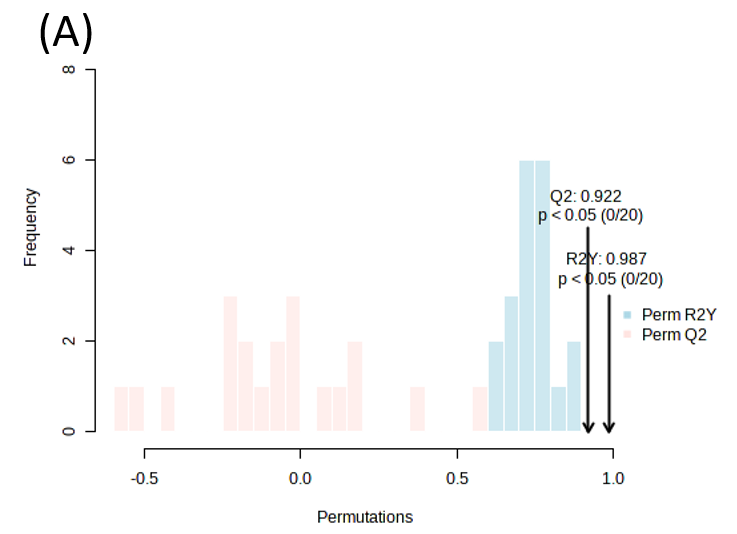
**


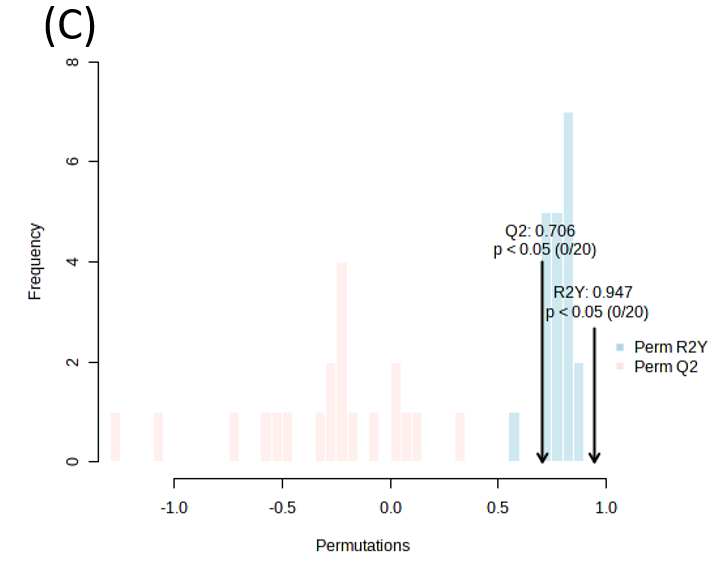


**Figure S2**: Frequency plot of 10 identified proteins **(A)** Endometrial Cancer and Control **(B)** Hyperplasia and Control **(C)** Endometrial Cancer and Hyperplasia

**
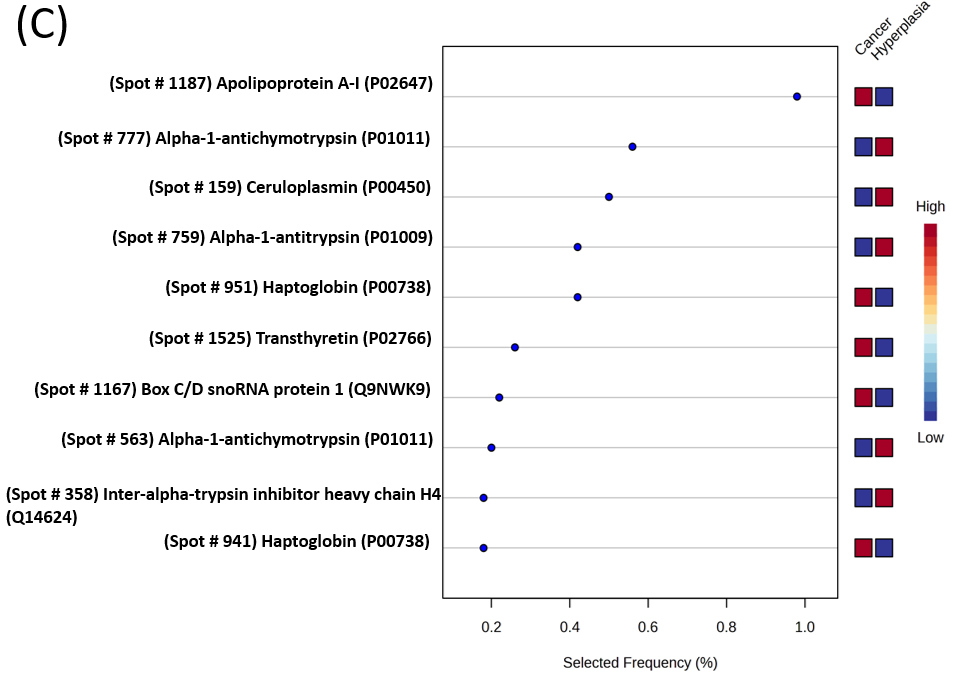

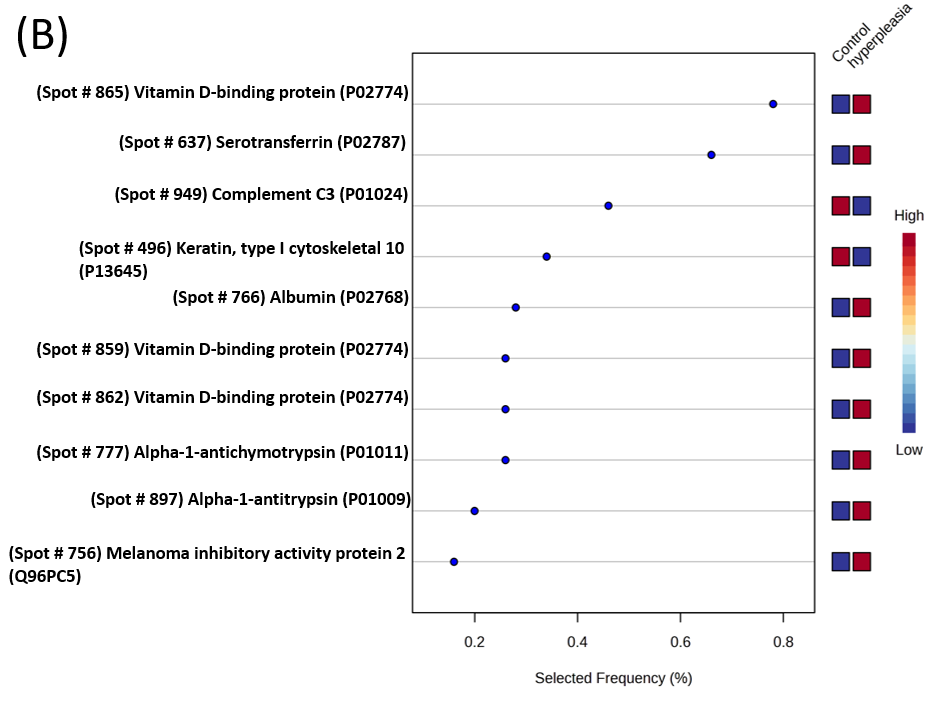

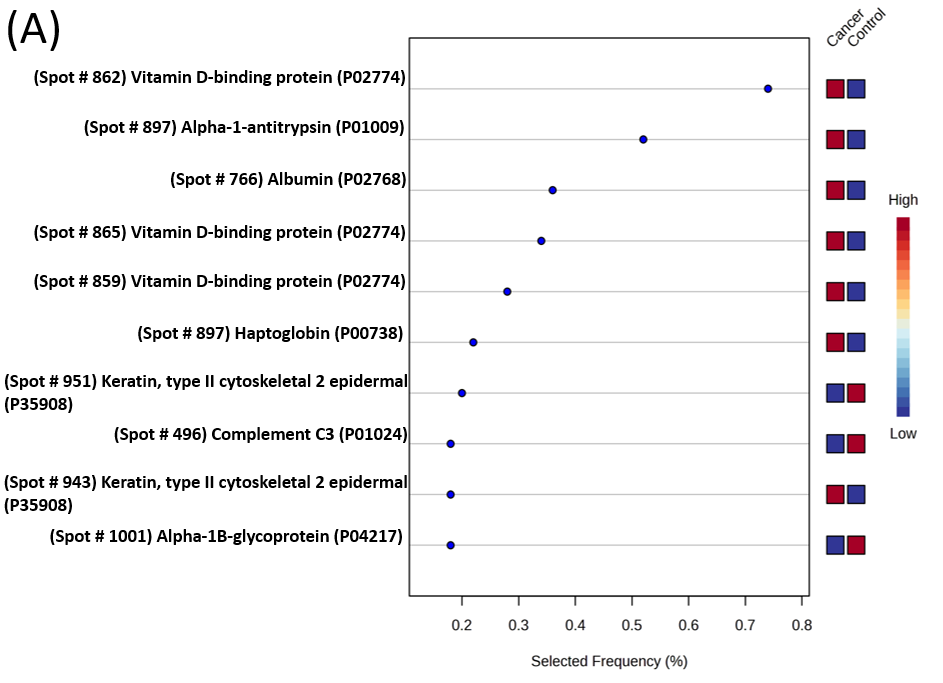
**

**
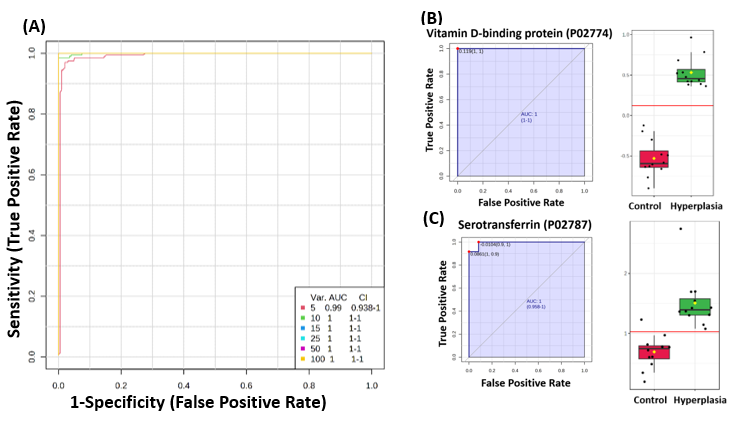
Figure S3:** Results of biomarker evaluation in Hyperplasia (Hy) and Controls (CO). (**A**) The Receiver Operating Characteristics (ROC) curve was generated by the OPLS-DA model, with Area Under the Curve (AUC) values calculated from the combination of 5, 10, 15, 25, 50, and 100 proteins. **(B, C)** Two upregulated proteins in hyperplasia with the highest AUC; (**B**) Vitamin D- binding protein, AUC = 1.00; Box plot (FDR p ≤ 0.05 and fold change ≥1.5), where red represents CO and green represents Hy; (**C**) Serotransferrin, AUC = 1.00; Box plot (FDR p ≤ 0.05 and fold change ≥1.5), where red represents CO and green represents Hy.

**Figure S4:** Results of biomarker evaluation in Endometrial cancer (EC) and Hyperplasia (Hy). (**A**) The Receiver Operating Characteristics (ROC) curve was generated by the OPLS-DA model, with Area Under the Curve (AUC) values calculated from the combination of 5, 10, 15, 25, 50, and 100 proteins (**B**) A downregulated protein in EC with the highest AUC, Alpha-1-antichymotrypsin, AUC = 0.95; Box plot (FDR p ≤ 0.05 and fold change ≥1.5), where red represents EC and green represents Hy. An upregulated protein in EC with the highest AUC, Apolipoprotein A-1, AUC = 0.908; Box plot (FDR p ≤ 0.05 and fold change ≥1.5), where red represents EC and green represents Hy.


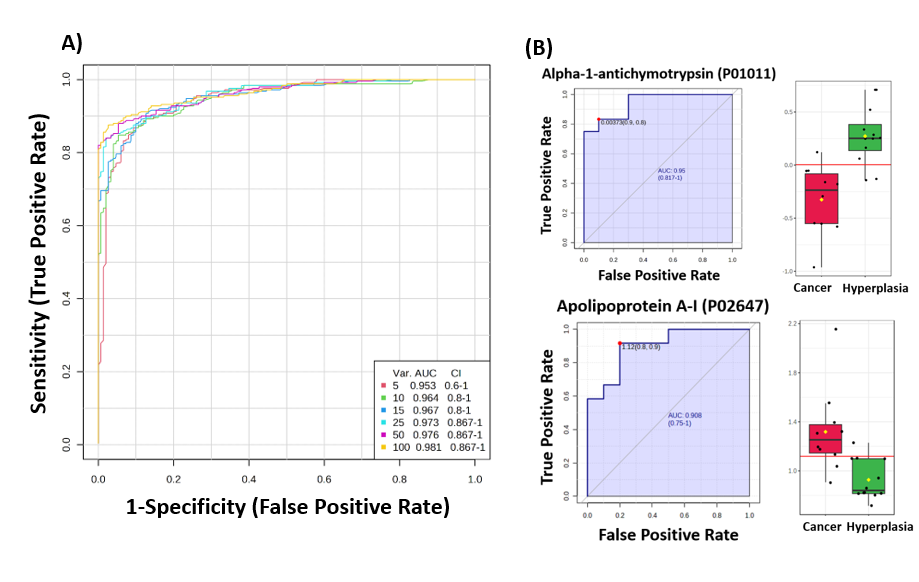


**Figure S5**: The most enriched interaction network of the differentially expressed proteins in different groups. Protein nodes with blue halo indicate up-regulated; protein nodes with red halo indicate down-regulated. Protein nodes without halo are proposed by STRING database and indicate potential targets that were functionally coordinated with the differentially expressed proteins. Solid black line indicates co-expression; green line indicates gene neighbourhood; dark blue line indicates gene co-occurrence; purple line indicates experimentally determined protein interactions. **(A)** Interaction network of differentially expressed proteins in the hyperplasia group compared to control group. Central nodes of the pathway related to signaling of complement and coagulation cascades, remodeling, and clearance, Regulation of insulin-like growth factor (IGF) transport and uptake by Insulin-like growth factor binding proteins (IGFBPs), hemostasis, regulation of complement cascade (KEGG, Reactome databases) were found to be dysregulated between the two states. **(B)** Interaction network of differentially expressed proteins in the endometrial cancer group compared to the hyperplasia group. Central nodes of the pathway related to signaling of complement and coagulation cascades, Regulation of insulin-like growth factor (IGF) transport and uptake by Insulin-like growth factor binding proteins (IGFBPs), cholesterol metabolism, platelet degranulation, platelet activation, signaling and aggregation, hemostasis (KEGG, Reactome databases) were found to be dysregulated between the two states.


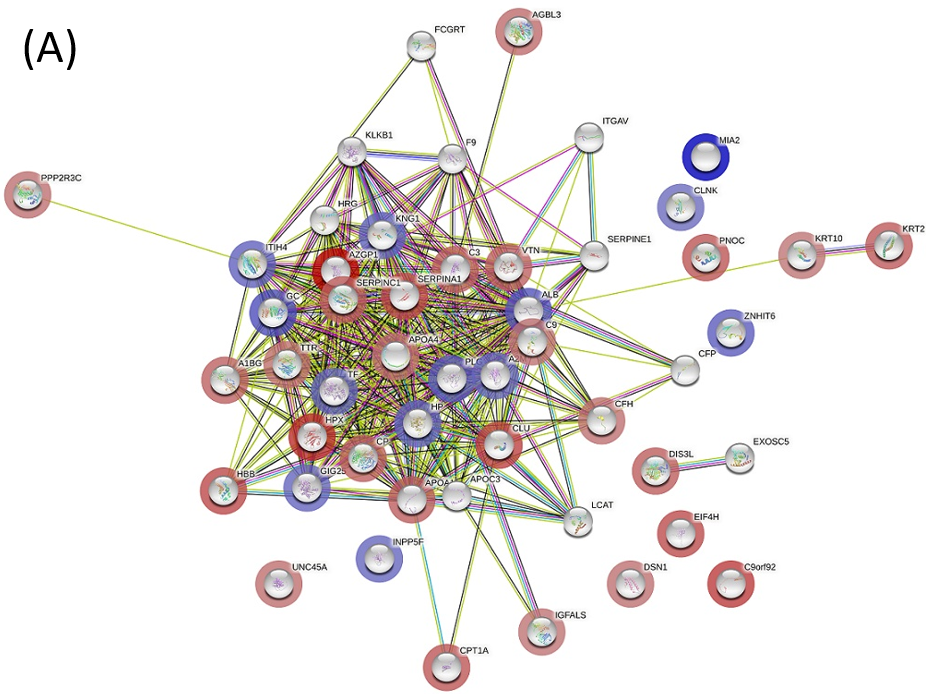


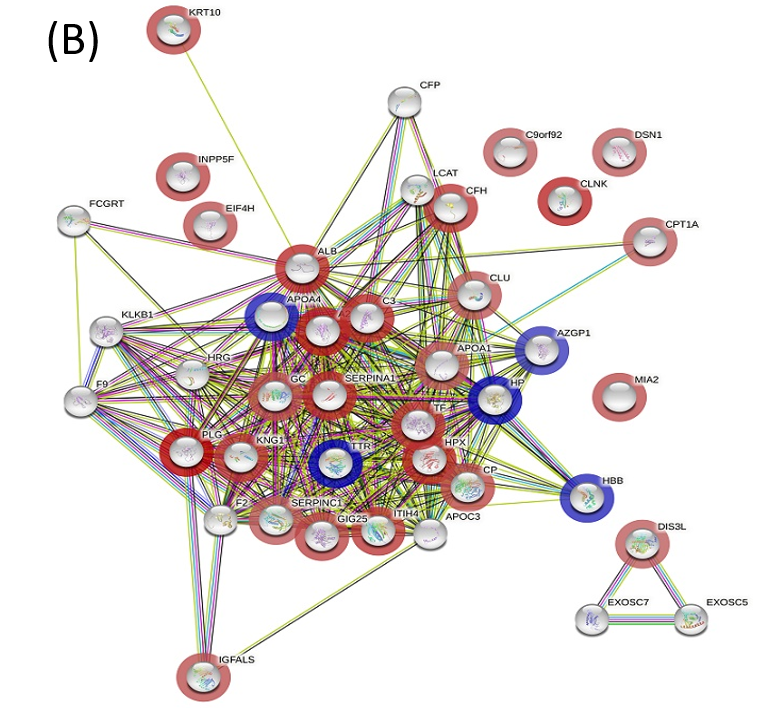


**Table S1:** Characteristics of study subjects

| **Sample ID** | **Sample type** | **Age** | **BMI** | **Diagnosis** | **Stage/Grade** |
| --- | --- | --- | --- | --- | --- |
| EC1 | Endometrial Cancer | 70 | 33.69 | Endometrioid endometrial adenocarcinoma | 1A/G2 |
| EC2 | Endometrial Cancer | 60 | 48.50 | Endometrioid endometrial adenocarcinoma | 1A/G2 |
| EC 3 | Endometrial Cancer | 60 | 48.00 | Endometrioid endometrial adenocarcinoma | 1A/G1 |
| EC 4 | Endometrial Cancer | 45 | 29.90 | Endometrioid endometrial adenocarcinoma | 1B/G3 |
| EC 5 | Endometrial Cancer | 50 | 32.00 | Endometrioid endometrial adenocarcinoma | 1B/G1 |
| EC 6 | Endometrial Cancer | 68 | 27.89 | Endometrioid endometrial adenocarcinoma | 3C/G3 |
| EC 7 | Endometrial Cancer | 50 | 31.00 | Endometrioid endometrial adenocarcinoma | 3C/G2 |
| EC 8 | Endometrial Cancer | 72 | 25.36 | Endometrioid endometrial adenocarcinoma | 3A/G2 |
| EC 9 | Endometrial Cancer | 59 | 45.61 | Endometrioid endometrial adenocarcinoma | 1A/G3 |
| EC 10 | Endometrial Cancer | 54 | 30.00 | Endometrioid endometrial adenocarcinoma | 1A/G2 |
| Hy1 | Hyperplasia | 49 | 26.03 | hyperplastic endometrium without atypia | |
| Hy2 | Hyperplasia | 49 | 32.00 | hyperplastic endometrium with atypia | |
| Hy3 | Hyperplasia | 48 | 33.30 | hyperplastic endometrium with atypia | |
| Hy4 | Hyperplasia | 48 | 34.20 | hyperplastic endometrium with atypia | |
| Hy5 | Hyperplasia | 48 | 34.00 | hyperplastic endometrium without atypia | |
| Hy6 | Hyperplasia | 58 | 39.90 | hyperplastic endometrium with atypia | |
| Hy7 | Hyperplasia | 65 | 38.20 | hyperplastic endometrium with atypia | |
| Hy8 | Hyperplasia | 65 | 37.90 | hyperplastic endometrium with atypia | |
| Hy9 | Hyperplasia | 58 | 37.93 | hyperplastic endometrium with atypia | |
| Hy10 | Hyperplasia | 61 | 48.13 | hyperplastic endometrium with atypia | |
| Hy11 | Hyperplasia | 50 | 36.03 | hyperplastic endometrium with atypia | |
| Hy12 | Hyperplasia | 59 | 38.01 | hyperplastic endometrium with atypia | |
| CO1 | Control | 48 | 28.21 | Normal endometrium | |
| CO 2 | Control | 43 | 39.89 | Normal endometrium | |
| CO 3 | Control | 63 | 41.62 | Normal endometrium | |
| CO 4 | Control | 44 | 46.93 | Normal endometrium | |
| CO 5 | Control | 65 | 30.02 | Normal endometrium | |
| CO6 | Control | 52 | 24.90 | Normal endometrium | |
| CO 7 | Control | 52 | 23.70 | Normal endometrium | |
| CO8 | Control | 52 | 23.30 | Normal endometrium | |
| CO 9 | Control | 43 | 39.90 | Normal endometrium | |
| CO 10 | Control | 43 | 39.80 | Normal endometrium | |
| CO 11 | Control | 40 | 39.02 | Normal endometrium | |
| CO 12 | Control | 43 | 29.20 | Normal endometrium | |

**Table S2:** Experimental design- 34 samples run on 17 2D-PAGE gels, samples were labeled randomly with Cy3 and Cy5, and a pooled sample was used as an internal standard and was stained with Cy2.

| **Cy2** | **Cy5** | **Cy3** | **Gel** |
| --- | --- | --- | --- |
| **Pooled sample** | **EC1** | **Hy1** | **1** |
| **Pooled sample** | **Hy2** | **CO1** | **2** |
| **Pooled sample** | **CO2** | **EC2** | **3** |
| **Pooled sample** | **EC3** | **Hy3** | **4** |
| **Pooled sample** | **Hy4** | **CO3** | **5** |
| **Pooled sample** | **CO4** | **EC4** | **6** |
| **Pooled sample** | **EC5** | **Hy5** | **7** |
| **Pooled sample** | **Hy6** | **CO5** | **8** |
| **Pooled sample** | **CO6** | **EC6** | **9** |
| **Pooled sample** | **EC7** | **Hy7** | **10** |
| **Pooled sample** | **Hy8** | **CO7** | **11** |
| **Pooled sample** | **CO8** | **EC8** | **12** |
| **Pooled sample** | **EC9** | **Hy9** | **13** |
| **Pooled sample** | **CO9** | **EC10** | **14** |
| **Pooled sample** | **Hy10** | **CO10** | **15** |
| **Pooled sample** | **CO11** | **Hy11** | **16** |
| **Pooled sample** | **Hy12** | **CO12** | **17** |
| **Hy: HYPERPLASIA; CO: CONTROL; EC: ENDOMETRIAL CANCER** | | | |

**Table S3:** Mass spectrometry list of significant differentially abundant proteins between endometrial cancer, hyperplasia and control states identified in plasma samples, using 2D-DIGE with. Protein name, accession number, Mascot score, MS % coverage, protein MW and pI values according to Uniprot database are listed.

| **Sl no:** | **Spot No^a^** | **Accession Nob** | **Protein Name** | **MASCOT ID** | **Pi^c^** | **MW^d^** | **Cov%** | **Score** |
| --- | --- | --- | --- | --- | --- | --- | --- | --- |
| 1 | 69 | Q8NCR6 | Spermatid-specific manchette-related protein 1 | CI024_HUMAN | 8.76 | 31002 | 39 | 57 |
| 2 | 116 | P08603 | Complement factor H | CFAH_HUMAN | 6.21 | 143680 | 25 | 127 |
| 3 | 159 | P00450 | Ceruloplasmin | CERU_HUMAN | 5.44 | 122983 | 19 | 69 |
| 4 | 283 | P00450 | Ceruloplasmin | CERU_HUMAN | 5.44 | 122983 | 33 | 156 |
| 5 | 303 | P00450 | Ceruloplasmin | CERU_HUMAN | 5.44 | 122983 | 36 | 169 |
| 6 | 316 | P00450 | Ceruloplasmin | CERU_HUMAN | 5.44 | 122983 | 19 | 69 |
| 7 | 323 | Q14624 | Inter-alpha-trypsin inhibitor heavy chain H4 | ITIH4_HUMAN | 6.51 | 103521 | 26 | 84 |
| 8 | 339 | P01042 | Kininogen-1 | KNG1_HUMAN | 6.43 | 72996 | 14 | 67 |
| 9 | 343 | P13645 | Keratin, type I cytoskeletal 10 | K1C10_HUMAN | 5.13 | 59020 | 13 | 59 |
| 10 | 358 | Q14624 | Inter-alpha-trypsin inhibitor heavy chain H4 | ITIH4_HUMAN | 6.51 | 103521 | 19 | 73 |
| 11 | 400 | P02787 | Serotransferrin | TRFE_HUMAN | 6.81 | 79280 | 35 | 153 |
| 12 | 402 | P02787 | Serotransferrin | TRFE_HUMAN | 6.81 | 79280 | 43 | 128 |
| 13 | 410 | P01042 | Kininogen-1 | KNG1_HUMAN | 6.43 | 72996 | 34 | 133 |
| 14 | 424 | P00747 | Plasminogen | PLMN_HUMAN | 7.04 | 93247 | 33 | 144 |
| 15 | 426 | P13645 | Keratin, type I cytoskeletal 10 | K1C10_HUMAN | 5.13 | 59020 | 16 | 63 |
| 16 | 429 | Q9Y2H2 | Phosphatidylinositide phosphatase SAC2 | SAC2_HUMAN | 6.57 | 129352 | 9 | 62 |
| 17 | 432 | P01042 | Kininogen-1 | KNG1_HUMAN | 6.43 | 72996 | 16 | 81 |
| 18 | 434 | P01023 | Alpha-2-macroglobulin | A2MG_HUMAN | 6 | 164614 | 19 | 69 |
| 19 | 466 | Q9NWK9 | Box C/D snoRNA protein 1 | BCD1_HUMAN | 5.62 | 54511 | 21 | 58 |
| 20 | 491 | P02787 | Serotransferrin | TRFE_HUMAN | 6.81 | 79280 | 24 | 65 |
| 21 | 496 | P13645 | Keratin, type I cytoskeletal 10 | K1C10_HUMAN | 5.13 | 59020 | 19 | 88 |
| 22 | 498 | P01023 | Alpha-2-macroglobulin | A2MG_HUMAN | 6 | 164614 | 22 | 95 |
| 23 | 509 | P02790 | Hemopexin | HEMO_HUMAN | 6.22 | 52385 | 35 | 147 |
| 24 | 516 | P04217 | Alpha-1B-glycoprotein | A1BG_HUMAN | 5.58 | 54809 | 32 | 117 |
| 25 | 517 | P10909 | Clusterin | CLUS_HUMAN | 5.89 | 53031 | 19 | 77 |
| 26 | 519 | P02748 | Complement component C9 | CO9_HUMAN | 5.43 | 64615 | 30 | 64 |
| 27 | 520 | P04217 | Alpha-1B-glycoprotein | A1BG_HUMAN | 5.58 | 54809 | 36 | 111 |
| 28 | 539 | P02768 | Albumin | ALBU_HUMAN | 5.92 | 71317 | 21 | 57 |
| 29 | 546 | P01011 | Alpha-1-antichymotrypsin | AACT_HUMAN | 5.33 | 47792 | 33 | 158 |
| 30 | 547 | P35858 | Insulin-like growth factor-binding protein complex acid labile subunit | ALS_HUMAN | 6.33 | 66735 | 24 | 59 |
| 31 | 551 | P01023 | Alpha-2-macroglobulin | A2MG_HUMAN | 6 | 164614 | 15 | 95 |
| 32 | 563 | P01011 | Alpha-1-antichymotrypsin | AACT_HUMAN | 5.33 | 47792 | 32 | 118 |
| 33 | 581 | P02787 | Serotransferrin | TRFE_HUMAN | 6.81 | 79280 | 37 | 147 |
| 34 | 588 | P02787 | Serotransferrin | TRFE_HUMAN | 6.81 | 79280 | 48 | 236 |
| 35 | 604 | P00450 | Ceruloplasmin | CERU_HUMAN | 5.44 | 122983 | 15 | 68 |
| 36 | 608 | Q14624 | Inter-alpha-trypsin inhibitor heavy chain H4 | ITIH4_HUMAN | 6.51 | 103521 | 26 | 84 |
| 37 | 613 | P01024 | Complement C3 | CO3_HUMAN | 6.02 | 188569 | 13 | 69 |
| 38 | 615 | P02787 | Serotransferrin | TRFE_HUMAN | 6.81 | 79280 | 17 | 66 |
| 39 | 623 | P02790 | Hemopexin | HEMO_HUMAN | 6.22 | 52385 | 42 | 150 |
| 40 | 624 | P02768 | Albumin | ALBU_HUMAN | 5.92 | 71317 | 29 | 69 |
| 41 | 626 | P02787 | Serotransferrin | TRFE_HUMAN | 6.81 | 79280 | 49 | 339 |
| 42 | 628 | P02790 | Hemopexin | HEMO_HUMAN | 6.22 | 52385 | 27 | 112 |
| 43 | 629 | Q7Z7G1 | Cytokine-dependent hematopoietic cell linker | CLNK_HUMAN | 9.11 | 49808 | 19 | 57 |
| 44 | 631 | P08603 | Complement factor H | CFAH_HUMAN | 6.21 | 143680 | 25 | 127 |
| 45 | 632 | P02790 | Hemopexin | HEMO_HUMAN | 6.22 | 52385 | 25 | 90 |
| 46 | 634 | P01008 | Antithrombin-III | ANT3_HUMAN | 6.32 | 53025 | 16 | 68 |
| 47 | 635 | P02787 | Serotransferrin | TRFE_HUMAN | 6.81 | 79280 | 43 | 164 |
| 48 | 636 | P02790 | Hemopexin | HEMO_HUMAN | 6.22 | 52385 | 43 | 118 |
| 49 | 637 | P02787 | Serotransferrin | TRFE_HUMAN | 6.81 | 79280 | 46 | 241 |
| 50 | 646 | P00450 | Ceruloplasmin | CERU_HUMAN | 5.44 | 122983 | 36 | 169 |
| 51 | 651 | P01009 | Alpha-1-antitrypsin | A1AT_HUMAN | 5.37 | 46878 | 26 | 88 |
| 52 | 657 | P02787 | Serotransferrin | TRFE_HUMAN | 6.81 | 79280 | 29 | 159 |
| 53 | 658 | P02787 | Serotransferrin | TRFE_HUMAN | 6.81 | 79280 | 30 | 121 |
| 54 | 666 | P01011 | Alpha-1-antichymotrypsin | AACT_HUMAN | 5.33 | 47792 | 32 | 118 |
| 55 | 668 | P01024 | Complement C3 | CO3_HUMAN | 6.02 | 188569 | 22 | 83 |
| 56 | 669 | P02787 | Serotransferrin | TRFE_HUMAN | 6.81 | 79280 | 29 | 159 |
| 57 | 673 | Q5W150 | Putative uncharacterized protein MGC163334 | YT011_HUMAN | 9.75 | 15542 | 22 | 59 |
| 58 | 690 | P01009 | Alpha-1-antitrypsin | A1AT_HUMAN | 5.37 | 46878 | 60 | 143 |
| 59 | 700 | P01008 | Antithrombin-III | ANT3_HUMAN | 6.32 | 53025 | 35 | 100 |
| 60 | 714 | P01008 | Antithrombin-III | ANT3_HUMAN | 6.32 | 53025 | 42 | 136 |
| 61 | 745 | P02768 | Albumin | ALBU_HUMAN | 5.92 | 71317 | 32 | 62 |
| 62 | 756 | Q96PC5 | Melanoma inhibitory activity protein 2 | CTGE5_HUMAN | 5.15 | 91197 | 15 | 57 |
| 63 | 759 | P01009 | Alpha-1-antitrypsin | A1AT_HUMAN | 5.37 | 46878 | 45 | 88 |
| 64 | 766 | P02768 | Albumin | ALBU_HUMAN | 5.92 | 71317 | 28 | 73 |
| 65 | 777 | P01011 | Alpha-1-antichymotrypsin | AACT_HUMAN | 5.33 | 47792 | 27 | 87 |
| 66 | 807 | P01023 | Alpha-2-macroglobulin | A2MG_HUMAN | 6 | 164614 | 23 | 124 |
| 67 | 827 | P35908 | Keratin, type II cytoskeletal 2 epidermal | K22E_HUMAN | 8.07 | 65678 | 17 | 57 |
| 68 | 830 | Q9H410 | Kinetochore-associated protein DSN1 homolog | DSN1_HUMAN | 6.58 | 40384 | 26 | 63 |
| 69 | 844 | P02774 | Vitamin D-binding protein | VTDB_HUMAN | 5.4 | 54526 | 46 | 126 |
| 70 | 858 | P01042 | Kininogen-1 | KNG1_HUMAN | 6.43 | 72996 | 34 | 73 |
| 71 | 859 | P02774 | Vitamin D-binding protein | VTDB_HUMAN | 5.4 | 54526 | 45 | 111 |
| 72 | 862 | P02774 | Vitamin D-binding protein | VTDB_HUMAN | 5.4 | 54526 | 34 | 88 |
| 73 | 865 | P02774 | Vitamin D-binding protein | VTDB_HUMAN | 5.4 | 54526 | 42 | 113 |
| 74 | 885 | P00738 | Haptoglobin | HPT_HUMAN | 6.13 | 45861 | 24 | 94 |
| 75 | 897 | P01009 | Alpha-1-antitrypsin | A1AT_HUMAN | 5.37 | 46878 | 45 | 144 |
| 76 | 900 | P00738 | Haptoglobin | HPT_HUMAN | 6.13 | 45861 | 33 | 98 |
| 77 | 903 | P02774 | Vitamin D-binding protein | VTDB_HUMAN | 5.4 | 54526 | 31 | 82 |
| 78 | 911 | Q8NEM8 | Cytosolic carboxypeptidase 3 | CBPC3_HUMAN | 8.98 | 117135 | 19 | 57 |
| 79 | 912 | P04004 | Vitronectin | VTNC_HUMAN | 5.55 | 55069 | 25 | 65 |
| 80 | 922 | P00738 | Haptoglobin | HPT_HUMAN | 6.13 | 45861 | 33 | 136 |
| 81 | 941 | P00738 | Haptoglobin | HPT_HUMAN | 6.13 | 45861 | 33 | 74 |
| 82 | 943 | P35908 | Keratin, type II cytoskeletal 2 epidermal | K22E_HUMAN | 8.07 | 65678 | 17 | 57 |
| 83 | 949 | P01024 | Complement C3 | CO3_HUMAN | 6.02 | 188569 | 13 | 84 |
| 84 | 950 | P25311 | Zinc-alpha-2-glycoprotein | ZA2G_HUMAN | 5.57 | 34079 | 38 | 70 |
| 85 | 951 | P00738 | Haptoglobin | HPT_HUMAN | 6.13 | 45861 | 37 | 148 |
| 86 | 959 | P00450 | Ceruloplasmin | CERU_HUMAN | 5.44 | 122983 | 26 | 138 |
| 87 | 969 | P00738 | Haptoglobin | HPT_HUMAN | 6.13 | 45861 | 36 | 106 |
| 88 | 978 | P06727 | Apolipoprotein A-IV | APOA4_HUMAN | 5.28 | 45371 | 50 | 206 |
| 89 | 989 | P01009 | Alpha-1-antitrypsin | A1AT_HUMAN | 5.37 | 46878 | 30 | 66 |
| 90 | 992 | Q9H3U1 | Protein unc-45 homolog A | UN45A_HUMAN | 5.8 | 104266 | 12 | 58 |
| 91 | 993 | Q14624 | Inter-alpha-trypsin inhibitor heavy chain H4 | ITIH4_HUMAN | 6.51 | 103521 | 31 | 146 |
| 92 | 1001 | P04217 | Alpha-1B-glycoprotein | A1BG_HUMAN | 5.58 | 54809 | 31 | 100 |
| 93 | 1005 | A6NGG3 | Putative uncharacterized protein C9orf92 | CI092_HUMAN | 9.6 | 8799 | 46 | 57 |
| 94 | 1008 | Q15056 | Eukaryotic translation initiation factor 4H | IF4H_HUMAN | 6.67 | 27425 | 25 | 58 |
| 95 | 1009 | Q13519 | Prepronociceptin | PNOC_HUMAN | 8.73 | 20681 | 31 | 64 |
| 96 | 1010 | P13645 | Keratin, type I cytoskeletal 10 | K1C10_HUMAN | 5.13 | 59020 | 19 | 60 |
| 97 | 1014 | Q8TF46 | DIS3-like exonuclease 1 | DI3L1_HUMAN | 6.09 | 122194 | 8 | 57 |
| 98 | 1026 | P01009 | Alpha-1-antitrypsin | A1AT_HUMAN | 5.37 | 46878 | 28 | 81 |
| 99 | 1031 | P00450 | Ceruloplasmin | CERU_HUMAN | 5.44 | 122983 | 36 | 153 |
| 100 | 1044 | P06727 | Apolipoprotein A-IV | APOA4_HUMAN | 5.28 | 45371 | 25 | 69 |
| 101 | 1049 | P00450 | Ceruloplasmin | CERU_HUMAN | 5.44 | 122983 | 15 | 68 |
| 102 | 1051 | P68871 | Hemoglobin subunit beta | HBB_HUMAN | 6.75 | 16102 | 36 | 58 |
| 103 | 1066 | P02790 | Hemopexin | HEMO_HUMAN | 6.22 | 52385 | 19 | 86 |
| 104 | 1076 | P00738 | Haptoglobin | HPT_HUMAN | 6.13 | 45861 | 33 | 87 |
| 105 | 1148 | P50416 | Carnitine O-palmitoyltransferase 1, liver isoform | CPT1A_HUMAN | 8.85 | 88995 | 14 | 60 |
| 106 | 1152 | P02647 | Apolipoprotein A-I | APOA1_HUMAN | 5.56 | 30759 | 43 | 110 |
| 107 | 1167 | Q9NWK9 | Box C/D snoRNA protein 1 | BCD1_HUMAN | 5.62 | 54511 | 21 | 58 |
| 108 | 1187 | P02647 | Apolipoprotein A-I | APOA1_HUMAN | 5.56 | 30759 | 53 | 148 |
| 109 | 1191 | P02647 | Apolipoprotein A-I | APOA1_HUMAN | 5.56 | 30759 | 43 | 101 |
| 110 | 1369 | Q969Q6 | Serine/threonine-protein phosphatase 2A regulatory subunit B'' subunit gamma | P2R3C_HUMAN | 5.07 | 53567 | 17 | 57 |
| 111 | 1375 | P02790 | Hemopexin | HEMO_HUMAN | 6.22 | 52385 | 19 | 64 |
| 112 | 1445 | P00738 | Haptoglobin | HPT_HUMAN | 6.13 | 45861 | 35 | 98 |
| 113 | 1486 | P68871 | Hemoglobin subunit beta | HBB_HUMAN | 6.75 | 16102 | 63 | 101 |
| 114 | 1525 | P02766 | Transthyretin | TTHY_HUMAN | 5.52 | 15991 | 69 | 119 |

**Table S4**: Identified proteins, with changes in abundance of significantly differentially abundant proteins between Cancer, Hyperplasia and control states in plasma samples. The average ratio between the two states, with their corresponding levels of fold changes (FC ≥1.5) and one-way ANOVA (p-value < 0.05) using 2D-DIGE. [Analysis type: MALDI-TOF; database: SwissProt; taxonomy: Homo sapiens].

| **A: Comparison between EC and CO** | | | | | | | |
| --- | --- | --- | --- | --- | --- | --- | --- |
| **Sl no:** | **Spot No^a^** | **Accession No** | **Protein Name** | MASCOT ID | **P value^b^** | **Ratio^c^ EC/CO** | **Exp^d^** |
|  |  |  |  |  | **(ANOVA)** |  |  |
| 1 | 865 | P02774 | Vitamin D-binding protein | VTDB_HUMAN | 1.19E-09 | 5.26 | UP |
| 2 | 862 | P02774 | Vitamin D-binding protein | VTDB_HUMAN | 1.61E-08 | 4.98 | UP |
| 3 | 903 | P02774 | Vitamin D-binding protein | VTDB_HUMAN | 5.25E-07 | 3.94 | UP |
| 4 | 900 | P00738 | Haptoglobin | HPT_HUMAN | 2.74E-07 | 3.3 | UP |
| 5 | 949 | P01024 | Complement C3 | CO3_HUMAN | 2.75E-07 | -3.05 | DOWN |
| 6 | 756 | Q96PC5 | Melanoma inhibitory activity protein 2 | CTGE5_HUMAN | 6.39E-08 | 2.92 | UP |
| 7 | 989 | P01009 | Alpha-1-antitrypsin | A1AT_HUMAN | 1.15E-04 | -2.82 | DOWN |
| 8 | 1026 | P01009 | Alpha-1-antitrypsin | A1AT_HUMAN | 1.96E-04 | -2.61 | DOWN |
| 9 | 885 | P00738 | Haptoglobin | HPT_HUMAN | 5.53E-08 | 2.67 | UP |
| 10 | 1001 | P04217 | Alpha-1B-glycoprotein | A1BG_HUMAN | 2.00E-05 | -2.5 | DOWN |
| 11 | 636 | P02790 | Hemopexin | HEMO_HUMAN | 7.59E-06 | -2.48 | DOWN |
| 12 | 517 | P10909 | Clusterin | CLUS_HUMAN | 2.79E-05 | -2.47 | DOWN |
| 13 | 1005 | A6NGG3 | Putative uncharacterized protein C9orf92 | CI092_HUMAN | 0.002 | -2.45 | DOWN |
| 14 | 1008 | Q15056 | Eukaryotic translation initiation factor 4H | IF4H_HUMAN | 0.003 | -2.43 | DOWN |
| 15 | 959 | P00450 | Ceruloplasmin | CERU_HUMAN | 0.009 | 2.37 | UP |
| 16 | 950 | P25311 | Zinc-alpha-2-glycoprotein | ZA2G_HUMAN | 1.47E-04 | -2.28 | DOWN |
| 17 | 993 | Q14624 | Inter-alpha-trypsin inhibitor heavy chain H4 | ITIH4_HUMAN | 0.026 | 2.21 | UP |
| 18 | 859 | P02774 | Vitamin D-binding protein | VTDB_HUMAN | 4.58E-08 | 2.19 | UP |
| 19 | 745 | P02768 | Albumin | ALBU_HUMAN | 1.00E-06 | 2.16 | UP |
| 20 | 634 | P01008 | Antithrombin-III | ANT3_HUMAN | 8.10E-05 | -2.15 | DOWN |
| 21 | 491 | P02787 | Serotransferrin | TRFE_HUMAN | 0.013 | 2.13 | UP |
| 22 | 651 | P01009 | Alpha-1-antitrypsin | A1AT_HUMAN | 3.98E-05 | -2.12 | DOWN |
| 23 | 668 | P01024 | Complement C3 | CO3_HUMAN | 1.24E-05 | -2.11 | DOWN |
| 24 | 941 | P00738 | Haptoglobin | HPT_HUMAN | 9.57E-04 | 2.11 | UP |
| 25 | 658 | P02787 | Serotransferrin | TRFE_HUMAN | 1.43E-04 | 2.1 | UP |
| 26 | 466 | Q9NWK9 | Box C/D snoRNA protein 1 | BCD1_HUMAN | 0.016 | 2.1 | UP |
| 27 | 1445 | P00738 | Haptoglobin | HPT_HUMAN | 0.039 | 2.08 | UP |
| 28 | 1148 | P50416 | Carnitine O-palmitoyltransferase 1, liver isoform | CPT1A_HUMAN | 0.04 | -2.01 | DOWN |
| 29 | 766 | P02768 | Albumin | ALBU_HUMAN | 9.03E-06 | 2 | UP |
| 30 | 690 | P01009 | Alpha-1-antitrypsin | A1AT_HUMAN | 8.79E-05 | -1.99 | DOWN |
| 31 | 520 | P04217 | Alpha-1B-glycoprotein | A1BG_HUMAN | 1.49E-05 | -1.97 | DOWN |
| 32 | 951 | P00738 | Haptoglobin | HPT_HUMAN | 0.016 | 1.89 | UP |
| 33 | 969 | P00738 | Haptoglobin | HPT_HUMAN | 0.012 | 1.86 | UP |
| 34 | 498 | P01023 | Alpha-2-macroglobulin | A2MG_HUMAN | 0.005 | 1.82 | UP |
| 35 | 339 | P01042 | Kininogen-1 | KNG1_HUMAN | 8.28E-05 | 1.81 | UP |
| 36 | 608 | Q14624 | Inter-alpha-trypsin inhibitor heavy chain H4 | ITIH4_HUMAN | 9.79E-04 | 1.81 | UP |
| 37 | 669 | P02787 | Serotransferrin | TRFE_HUMAN | 0.006 | 1.8 | UP |
| 38 | 844 | P02774 | Vitamin D-binding protein | VTDB_HUMAN | 1.30E-04 | 1.78 | UP |
| 39 | 551 | P01023 | Alpha-2-macroglobulin | A2MG_HUMAN | 0.005 | 1.73 | UP |
| 40 | 1167 | Q9NWK9 | Box C/D snoRNA protein 1 | BCD1_HUMAN | 0.007 | 1.69 | UP |
| 41 | 604 | P00450 | Ceruloplasmin | CERU_HUMAN | 2.96E-04 | 1.68 | UP |
| 42 | 539 | P02768 | Albumin | ALBU_HUMAN | 0.014 | 1.68 | UP |
| 43 | 410 | P01042 | Kininogen-1 | KNG1_HUMAN | 0.002 | 1.67 | UP |
| 44 | 657 | P02787 | Serotransferrin | TRFE_HUMAN | 0.004 | 1.66 | UP |
| 45 | 581 | P02787 | Serotransferrin | TRFE_HUMAN | 0.019 | 1.64 | UP |
| 46 | 283 | P00450 | Ceruloplasmin | CERU_HUMAN | 2.50E-04 | 1.62 | UP |
| 47 | 303 | P00450 | Ceruloplasmin | CERU_HUMAN | 0.003 | 1.62 | UP |
| 48 | 637 | P02787 | Serotransferrin | TRFE_HUMAN | 0.001 | 1.59 | UP |
| 49 | 400 | P02787 | Serotransferrin | TRFE_HUMAN | 0.011 | 1.58 | UP |
| 50 | 316 | P00450 | Ceruloplasmin | CERU_HUMAN | 0.002 | 1.55 | UP |
| 51 | 402 | P02787 | Serotransferrin | TRFE_HUMAN | 0.035 | 1.54 | UP |
| 52 | 69 | Q8NCR6 | Spermatid-specific manchette-related protein 1 | CI024_HUMAN | 0.023 | 1.52 | UP |
| 53 | 1014 | Q8TF46 | DIS3-like exonuclease 1 | DI3L1_HUMAN | 0.017 | -1.87 | DOWN |
| 54 | 827 | P35908 | Keratin, type II cytoskeletal 2 epidermal | K22E_HUMAN | 0.004 | -1.8 | DOWN |
| 55 | 509 | P02790 | Hemopexin | HEMO_HUMAN | 0.002 | -1.79 | DOWN |
| 56 | 1066 | P02790 | Hemopexin | HEMO_HUMAN | 0.014 | -1.77 | DOWN |
| 57 | 1009 | Q13519 | Prepronociceptin | PNOC_HUMAN | 0.009 | -1.76 | DOWN |
| 58 | 714 | P01008 | Antithrombin-III | ANT3_HUMAN | 0.008 | -1.75 | DOWN |
| 59 | 516 | P04217 | Alpha-1B-glycoprotein | A1BG_HUMAN | 8.21E-05 | -1.71 | DOWN |
| 60 | 700 | P01008 | Antithrombin-III | ANT3_HUMAN | 0.003 | -1.7 | DOWN |
| 61 | 1191 | P02647 | Apolipoprotein A-I | APOA1_HUMAN | 0.046 | -1.63 | DOWN |
| 62 | 628 | P02790 | Hemopexin | HEMO_HUMAN | 0.005 | -1.62 | DOWN |
| 63 | 547 | P35858 | Insulin-like growth factor-binding protein complex acid labile subunit | ALS_HUMAN | 0.046 | -1.62 | DOWN |
| 64 | 424 | P00747 | Plasminogen | PLMN_HUMAN | 0.044 | -1.59 | DOWN |
| 65 | 912 | P04004 | Vitronectin | VTNC_HUMAN | 0.016 | -1.58 | DOWN |
| 66 | 1369 | Q969Q6 | Serine/threonine-protein phosphatase 2A regulatory subunit B'' subunit gamma | P2R3C_HUMAN | 0.039 | -1.58 | DOWN |
| 67 | 629 | Q7Z7G1 | Cytokine-dependent hematopoietic cell linker | CLNK_HUMAN | 0.014 | -1.57 | DOWN |
| 68 | 519 | P02748 | Complement component C9 | CO9_HUMAN | 0.029 | -1.57 | DOWN |
| 69 | 426 | P13645 | Keratin, type I cytoskeletal 10 | K1C10_HUMAN | 0.025 | -1.56 | DOWN |
| 70 | 631 | P08603 | Complement factor H | CFAH_HUMAN | 0.035 | -1.56 | DOWN |
| 71 | 546 | P01011 | Alpha-1-antichymotrypsin | AACT_HUMAN | 0.008 | -1.54 | DOWN |
| 72 | 496 | P13645 | Keratin, type I cytoskeletal 10 | K1C10_HUMAN | 0.033 | -1.54 | DOWN |
| 73 | 673 | Q5W150 | Putative uncharacterized protein MGC163334 | YT011_HUMAN | 0.047 | -1.54 | DOWN |
| 74 | 116 | P08603 | Complement factor H | CFAH_HUMAN | 0.011 | -1.52 | DOWN |
| 75 | 1375 | P02790 | Hemopexin | HEMO_HUMAN | 0.014 | -1.52 | DOWN |
| 76 | 830 | Q9H410 | Kinetochore-associated protein DSN1 homolog | DSN1_HUMAN | 0.026 | -1.52 | DOWN |
| 77 | 1044 | P06727 | Apolipoprotein A-IV | APOA4_HUMAN | 0.038 | -1.52 | DOWN |
| 78 | 1010 | P13645 | Keratin, type I cytoskeletal 10 | K1C10_HUMAN | 0.008 | -1.51 | DOWN |
| 79 | 623 | P02790 | Hemopexin | HEMO_HUMAN | 0.024 | -1.51 | DOWN |
| 80 | 613 | P01024 | Complement C3 | CO3_HUMAN | 0.033 | -1.51 | DOWN |
| 81 | 1152 | P02647 | Apolipoprotein A-I | APOA1_HUMAN | 0.048 | -1.51 | DOWN |
| 82 | 911 | Q8NEM8 | Cytosolic carboxypeptidase 3 | CBPC3_HUMAN | 0.022 | -1.5 | DOWN |
| 83 | 1051 | P68871 | Hemoglobin subunit beta | HBB_HUMAN | 0.05 | -1.5 | DOWN |
| 84 | 897 | P01009 | Alpha-1-antitrypsin | A1AT_HUMAN | 2.96E-07 | 3.94 | UP |
| 85 | 943 | P35908 | Keratin, type II cytoskeletal 2 epidermal | K22E_HUMAN | 0.003 | 1.8 | UP |
| B: Comparison between Hy and CO | | | | | | | |
| **Sl no:** | **Spot No^a^** | **Accession No** | **Protein Name** | **MASCOT ID** | **P value^b^** | **Ratio^c^ HY/CO** | **Exp^d^** |
|  |  |  |  |  | **(ANOVA)** |  |  |
| 1 | 865 | P02774 | Vitamin D-binding protein | VTDB_HUMAN | 1.19E-09 | 6.28 | UP |
| 2 | 862 | P02774 | Vitamin D-binding protein | VTDB_HUMAN | 1.61E-08 | 5.64 | UP |
| 3 | 859 | P02774 | Vitamin D-binding protein | VTDB_HUMAN | 4.58E-08 | 3.04 | UP |
| 4 | 885 | P00738 | Haptoglobin | HPT_HUMAN | 5.53E-08 | 2.44 | UP |
| 5 | 756 | Q96PC5 | Melanoma inhibitory activity protein 2 | CTGE5_HUMAN | 6.39E-08 | 3.43 | UP |
| 6 | 900 | P00738 | Haptoglobin | HPT_HUMAN | 2.74E-07 | 2.62 | UP |
| 7 | 949 | P01024 | Complement C3 | CO3_HUMAN | 2.75E-07 | -3.59 | DOWN |
| 8 | 903 | P02774 | Vitamin D-binding protein | VTDB_HUMAN | 5.25E-07 | 4.32 | UP |
| 9 | 745 | P02768 | Albumin | ALBU_HUMAN | 1.00E-06 | 2.51 | UP |
| 10 | 636 | P02790 | Hemopexin | HEMO_HUMAN | 7.59E-06 | -2.18 | DOWN |
| 11 | 635 | P02787 | Serotransferrin | TRFE_HUMAN | 8.32E-06 | 1.62 | UP |
| 12 | 766 | P02768 | Albumin | ALBU_HUMAN | 9.03E-06 | 2.34 | UP |
| 13 | 668 | P01024 | Complement C3 | CO3_HUMAN | 1.24E-05 | -1.72 | DOWN |
| 14 | 520 | P04217 | Alpha-1B-glycoprotein | A1BG_HUMAN | 1.49E-05 | -2.04 | DOWN |
| 15 | 1001 | P04217 | Alpha-1B-glycoprotein | A1BG_HUMAN | 2.00E-05 | -2.91 | DOWN |
| 16 | 517 | P10909 | Clusterin | CLUS_HUMAN | 2.79E-05 | -2.26 | DOWN |
| 17 | 651 | P01009 | Alpha-1-antitrypsin | A1AT_HUMAN | 3.98E-05 | -2.0 | DOWN |
| 18 | 634 | P01008 | Antithrombin-III | ANT3_HUMAN | 8.10E-05 | -1.83 | DOWN |
| 19 | 516 | P04217 | Alpha-1B-glycoprotein | A1BG_HUMAN | 8.21E-05 | -1.73 | DOWN |
| 20 | 339 | P01042 | Kininogen-1 | KNG1_HUMAN | 8.28E-05 | 1.54 | UP |
| 21 | 690 | P01009 | Alpha-1-antitrypsin | A1AT_HUMAN | 8.79E-05 | -1.79 | DOWN |
| 22 | 989 | P01009 | Alpha-1-antitrypsin | A1AT_HUMAN | 1.15E-04 | -3.06 | DOWN |
| 23 | 844 | P02774 | Vitamin D-binding protein | VTDB_HUMAN | 1.30E-04 | 2.07 | UP |
| 24 | 658 | P02787 | Serotransferrin | TRFE_HUMAN | 1.43E-04 | 1.88 | UP |
| 25 | 950 | P25311 | Zinc-alpha-2-glycoprotein | ZA2G_HUMAN | 1.47E-04 | -3.43 | DOWN |
| 26 | 1026 | P01009 | Alpha-1-antitrypsin | A1AT_HUMAN | 1.96E-04 | -2.12 | DOWN |
| 27 | 858 | P01042 | Kininogen-1 | KNG1_HUMAN | 7.27E-04 | 2.04 | UP |
| 28 | 941 | P00738 | Haptoglobin | HPT_HUMAN | 9.57E-04 | 1.68 | UP |
| 29 | 608 | Q14624 | Inter-alpha-trypsin inhibitor heavy chain H4 | ITIH4_HUMAN | 9.79E-04 | 2.06 | UP |
| 30 | 637 | P02787 | Serotransferrin | TRFE_HUMAN | 0.001 | 1.59 | UP |
| 31 | 777 | P01011 | Alpha-1-antichymotrypsin | AACT_HUMAN | 0.001 | 1.61 | UP |
| 32 | 646 | P00450 | Ceruloplasmin | CERU_HUMAN | 0.001 | -1.66 | DOWN |
| 33 | 509 | P02790 | Hemopexin | HEMO_HUMAN | 0.002 | -1.53 | DOWN |
| 34 | 1005 | A6NGG3 | Putative uncharacterized protein C9orf92 | CI092_HUMAN | 0.002 | -2.36 | DOWN |
| 35 | 1049 | P00450 | Ceruloplasmin | CERU_HUMAN | 0.002 | -2.44 | DOWN |
| 36 | 410 | P01042 | Kininogen-1 | KNG1_HUMAN | 0.002 | 1.66 | UP |
| 37 | 323 | Q14624 | Inter-alpha-trypsin inhibitor heavy chain H4 | ITIH4_HUMAN | 0.003 | 1.71 | UP |
| 38 | 1008 | Q15056 | Eukaryotic translation initiation factor 4H | IF4H_HUMAN | 0.003 | -2.09 | DOWN |
| 39 | 624 | P02768 | Albumin | ALBU_HUMAN | 0.003 | 2.25 | UP |
| 40 | 700 | P01008 | Antithrombin-III | ANT3_HUMAN | 0.003 | -1.59 | DOWN |
| 41 | 657 | P02787 | Serotransferrin | TRFE_HUMAN | 0.004 | 1.64 | UP |
| 42 | 827 | P35908 | Keratin, type II cytoskeletal 2 epidermal | K22E_HUMAN | 0.004 | -1.84 | DOWN |
| 43 | 498 | P01023 | Alpha-2-macroglobulin | A2MG_HUMAN | 0.005 | 1.68 | UP |
| 44 | 551 | P01023 | Alpha-2-macroglobulin | A2MG_HUMAN | 0.005 | 1.81 | UP |
| 45 | 669 | P02787 | Serotransferrin | TRFE_HUMAN | 0.006 | 1.93 | UP |
| 46 | 1010 | P13645 | Keratin, type I cytoskeletal 10 | K1C10_HUMAN | 0.008 | -1.68 | DOWN |
| 47 | 714 | P01008 | Antithrombin-III | ANT3_HUMAN | 0.008 | -1.75 | DOWN |
| 48 | 1009 | Q13519 | Prepronociceptin | PNOC_HUMAN | 0.009 | -1.93 | DOWN |
| 49 | 491 | P02787 | Serotransferrin | TRFE_HUMAN | 0.013 | 1.69 | UP |
| 50 | 539 | P02768 | Albumin | ALBU_HUMAN | 0.014 | 1.94 | UP |
| 51 | 629 | Q7Z7G1 | Cytokine-dependent hematopoietic cell linker | CLNK_HUMAN | 0.014 | 1.51 | UP |
| 52 | 1066 | P02790 | Hemopexin | HEMO_HUMAN | 0.014 | -2.61 | DOWN |
| 53 | 807 | P01023 | Alpha-2-macroglobulin | A2MG_HUMAN | 0.015 | 1.69 | UP |
| 54 | 466 | Q9NWK9 | Box C/D snoRNA protein 1 | BCD1_HUMAN | 0.016 | 1.82 | UP |
| 55 | 912 | P04004 | Vitronectin | VTNC_HUMAN | 0.016 | -1.72 | DOWN |
| 56 | 1031 | P00450 | Ceruloplasmin | CERU_HUMAN | 0.017 | -1.7 | DOWN |
| 57 | 1014 | Q8TF46 | DIS3-like exonuclease 1 | DI3L1_HUMAN | 0.017 | -1.77 | DOWN |
| 58 | 992 | Q9H3U1 | Protein unc-45 homolog A | UN45A_HUMAN | 0.017 | -1.58 | DOWN |
| 59 | 581 | P02787 | Serotransferrin | TRFE_HUMAN | 0.019 | 1.62 | UP |
| 60 | 1525 | P02766 | Transthyretin | TTHY_HUMAN | 0.02 | -1.57 | DOWN |
| 61 | 911 | Q8NEM8 | Cytosolic carboxypeptidase 3 | CBPC3_HUMAN | 0.022 | -1.55 | DOWN |
| 62 | 615 | P02787 | Serotransferrin | TRFE_HUMAN | 0.023 | 1.62 | UP |
| 63 | 666 | P01011 | Alpha-1-antichymotrypsin | AACT_HUMAN | 0.026 | 1.65 | UP |
| 64 | 830 | Q9H410 | Kinetochore-associated protein DSN1 homolog | DSN1_HUMAN | 0.026 | -1.56 | DOWN |
| 65 | 519 | P02748 | Complement component C9 | CO9_HUMAN | 0.029 | -1.51 | DOWN |
| 66 | 631 | P08603 | Complement factor H | CFAH_HUMAN | 0.035 | -1.56 | DOWN |
| 67 | 432 | P01042 | Kininogen-1 | KNG1_HUMAN | 0.035 | 1.71 | UP |
| 68 | 1044 | P06727 | Apolipoprotein A-IV | APOA4_HUMAN | 0.038 | -1.53 | DOWN |
| 69 | 1369 | Q969Q6 | Serine/threonine-protein phosphatase 2A regulatory subunit B'' subunit gamma | P2R3C_HUMAN | 0.039 | -1.53 | DOWN |
| 70 | 1148 | P50416 | Carnitine O-palmitoyltransferase 1, liver isoform | CPT1A_HUMAN | 0.04 | -1.91 | DOWN |
| 71 | 434 | P01023 | Alpha-2-macroglobulin | A2MG_HUMAN | 0.041 | 1.64 | UP |
| 72 | 424 | P00747 | Plasminogen | PLMN_HUMAN | 0.044 | 1.82 | UP |
| 73 | 1191 | P02647 | Apolipoprotein A-I | APOA1_HUMAN | 0.046 | -1.51 | DOWN |
| 74 | 1152 | P02647 | Apolipoprotein A-I | APOA1_HUMAN | 0.048 | -1.77 | DOWN |
| 75 | 429 | Q9Y2H2 | Phosphatidylinositide phosphatase SAC2 | SAC2_HUMAN | 0.048 | 1.64 | UP |
| 76 | 1486 | P68871 | Hemoglobin subunit beta | HBB_HUMAN | 0.049 | -1.59 | DOWN |
| 77 | 1051 | P68871 | Hemoglobin subunit beta | HBB_HUMAN | 0.05 | -2.12 | DOWN |
| 78 | 496 | P13645 | Keratin, type I cytoskeletal 10 | K1C10_HUMAN | 0.033 | -1.53 | DOWN |
| 79 | 862 | P02774 | Vitamin D-binding protein | VTDB_HUMAN | 1.61E-08 | 5.64 | UP |
| 80 | 897 | P01009 | Alpha-1-antitrypsin | A1AT_HUMAN | 2.96E-07 | -3.67 | DOWN |
| 81 | 756 | Q96PC5 | Melanoma inhibitory activity protein 2 | CTGE5_HUMAN | 6.39E-08 | 3.43 | UP |
| **C: Comparison between EC and Hy** | | | | | | | |
| **Sl no:** | **Spot No^a^** | **Accession No** | **Protein Name** | **MASCOT ID** | **P value^b^** | **Ratio^c^ EC/HY** | **Exp^d^** |
|  |  |  |  |  | **(ANOVA)** |  |  |
| 1 | 950 | P25311 | Zinc-alpha-2-glycoprotein | ZA2G_HUMAN | 1.47E-04 | 1.51 | UP |
| 2 | 159 | P00450 | Ceruloplasmin | CERU_HUMAN | 2.26E-04 | -1.5 | DOWN |
| 3 | 858 | P01042 | Kininogen-1 | KNG1_HUMAN | 7.27E-04 | -1.51 | DOWN |
| 4 | 777 | P01011 | Alpha-1-antichymotrypsin | AACT_HUMAN | 0.001 | -1.68 | DOWN |
| 5 | 759 | P01009 | Alpha-1-antitrypsin | A1AT_HUMAN | 0.001 | -1.55 | DOWN |
| 6 | 624 | P02768 | Albumin | ALBU_HUMAN | 0.003 | -1.53 | DOWN |
| 7 | 632 | P02790 | Hemopexin | HEMO_HUMAN | 0.005 | -1.8 | DOWN |
| 8 | 628 | P02790 | Hemopexin | HEMO_HUMAN | 0.005 | -1.52 | DOWN |
| 9 | 588 | P02787 | Serotransferrin | TRFE_HUMAN | 0.008 | -1.93 | DOWN |
| 10 | 116 | P08603 | Complement factor H | CFAH_HUMAN | 0.011 | -1.5 | DOWN |
| 11 | 969 | P00738 | Haptoglobin | HPT_HUMAN | 0.012 | 1.65 | UP |
| 12 | 1076 | P00738 | Haptoglobin | HPT_HUMAN | 0.012 | 1.57 | UP |
| 13 | 629 | Q7Z7G1 | Cytokine-dependent hematopoietic cell linker | CLNK_HUMAN | 0.014 | -1.62 | DOWN |
| 14 | 1525 | P02766 | Transthyretin | TTHY_HUMAN | 0.02 | 2.15 | UP |
| 15 | 626 | P02787 | Serotransferrin | TRFE_HUMAN | 0.023 | -1.58 | DOWN |
| 16 | 623 | P02790 | Hemopexin | HEMO_HUMAN | 0.024 | -1.61 | DOWN |
| 17 | 343 | P13645 | Keratin, type I cytoskeletal 10 | K1C10_HUMAN | 0.024 | -1.5 | DOWN |
| 18 | 426 | P13645 | Keratin, type I cytoskeletal 10 | K1C10_HUMAN | 0.025 | -1.72 | DOWN |
| 19 | 613 | P01024 | Complement C3 | CO3_HUMAN | 0.033 | -1.56 | DOWN |
| 20 | 432 | P01042 | Kininogen-1 | KNG1_HUMAN | 0.035 | -1.55 | DOWN |
| 21 | 922 | P00738 | Haptoglobin | HPT_HUMAN | 0.037 | 2.03 | UP |
| 22 | 358 | Q14624 | Inter-alpha-trypsin inhibitor heavy chain H4 | ITIH4_HUMAN | 0.037 | -1.52 | DOWN |
| 23 | 978 | P06727 | Apolipoprotein A-IV | APOA4_HUMAN | 0.038 | 1.81 | UP |
| 24 | 434 | P01023 | Alpha-2-macroglobulin | A2MG_HUMAN | 0.041 | -1.7 | DOWN |
| 25 | 424 | P00747 | Plasminogen | PLMN_HUMAN | 0.044 | -1.99 | DOWN |
| 26 | 673 | Q5W150 | Putative uncharacterized protein MGC163334 | YT011_HUMAN | 0.047 | -1.53 | DOWN |
| 27 | 1486 | P68871 | Hemoglobin subunit beta | HBB_HUMAN | 0.049 | 2.04 | UP |
| 28 | 1051 | P68871 | Hemoglobin subunit beta | HBB_HUMAN | 0.05 | 1.71 | UP |
| 29 | 1187 | P02647 | Apolipoprotein A-I | APOA1_HUMAN | 0.003 | 1.52 | UP |
| 30 | 951 | P00738 | Haptoglobin | HPT_HUMAN | 0.016 | 1.53 | UP |
| 31 | 1167 | Q9NWK9 | Box C/D snoRNA protein 1 | BCD1_HUMAN | 0.007 | 1.58 | UP |
| 32 | 563 | P01011 | Alpha-1-antichymotrypsin | AACT_HUMAN | 0.011 | -1.56 | DOWN |
| 33 | 941 | P00738 | Haptoglobin | HPT_HUMAN | 9.57E-04 | 1.55 | UP |

^a^ Protein accession number for SWISSPROT database.

^b^ p-Value (ANOVA).

^c^ Ratio between the groups.

^d^ Protein expression between the groups.

**Table S5:** The table shows the different canonical pathways obtained from STRING database analysis**. (A)** EC and CO group **(B)** Hy and CO group **(C)** EC and Hy group

| **(A) EC and CO** | | | | | | | | | | | | |  |
| --- | --- | --- | --- | --- | --- | --- | --- | --- | --- | --- | --- | --- | --- |
| **#term ID** | **term description** | | **observed gene count** | | **background gene count** | | **strength** | | **false discovery rate** | | **matching proteins in your network (labels)** | |  |
| hsa04610 | Complement and coagulation cascades | | 10 | | 82 | | 1.79 | | 5.98E-13 | | VTN,C3,C9,KNG1,PLG,CLU,A2M,CFH,SERPINC1,SERPINA1 | |  |
| HSA-381426 | Regulation of Insulin-like Growth Factor (IGF) transport and uptake by Insulin-like Growth Factor Binding Proteins (IGFBPs) | | 10 | | 124 | | 1.61 | | 6.16E-11 | | APOA1,C3,CP,KNG1,ALB,PLG,SERPINC1,TF,SERPINA1,IGFALS | |  |
| HSA-8957275 | Post-translational protein phosphorylation | | 8 | | 107 | | 1.57 | | 2.52E-08 | | APOA1,C3,CP,KNG1,ALB,SERPINC1,TF,SERPINA1 | |  |
| HSA-109582 | Hemostasis | | 13 | | 605 | | 1.03 | | 3.71E-08 | | APOA1,A1BG,KNG1,ITIH4,ALB,PLG,CLU,A2M,HBB,SERPINC1,GIG25,TF,SERPINA1 | |  |
| HSA-2168880 | Scavenging of heme from plasma | | 5 | | 13 | | 2.29 | | 6.23E-08 | | APOA1,HPX,ALB,HBB,HP | |  |
| HSA-977606 | Regulation of Complement cascade | | 5 | | 47 | | 1.73 | | 1.40E-05 | | VTN,C3,C9,CLU,CFH | |  |
| HSA-168249 | Innate Immune System | | 11 | | 1025 | | 0.73 | | 0.00072 | | VTN,C3,A1BG,C9,CLU,HBB,HP,CFH,GIG25,DSN1,SERPINA1 | |  |
| HSA-8963898 | Plasma lipoprotein assembly | | 3 | | 19 | | 1.9 | | 0.002 | | APOA1,A2M,APOA4 | |  |
| HSA-174824 | Plasma lipoprotein assembly, remodeling, and clearance | | 4 | | 71 | | 1.45 | | 0.0025 | | APOA1,ALB,A2M,APOA4 | |  |
| HSA-140837 | Intrinsic Pathway of Fibrin Clot Formation | | 3 | | 23 | | 1.82 | | 0.0028 | | KNG1,A2M,SERPINC1 | |  |
| HSA-6798695 | Neutrophil degranulation | | 7 | | 473 | | 0.87 | | 0.0057 | | C3,A1BG,HBB,HP,GIG25,DSN1,SERPINA1 | |  |
| HSA-8963899 | Plasma lipoprotein remodeling | | 3 | | 32 | | 1.67 | | 0.0062 | | APOA1,ALB,APOA4 | |  |
| **(B) Hy and CO** | | | | | | | | | | | | |  |
| **#term ID** | **term description** | | **observed gene count** | | **background gene count** | | **strength** | | **false discovery rate** | | **matching proteins in your network (labels)** | |  |
| hsa04610 | Complement and coagulation cascades | | 13 | | 82 | | 1.79 | | 3.54E-17 | | F9,SERPINE1,VTN,C3,C9,KLKB1,KNG1,PLG,CLU,A2M,CFH,SERPINC1,SERPINA1 | |  |
| hsa04979 | Cholesterol metabolism | | 4 | | 48 | | 1.51 | | 0.0016 | | APOC3,APOA1,LCAT,APOA4 | |  |
| HSA-109582 | Hemostasis | | 18 | | 605 | | 1.07 | | 4.30E-12 | | F9,SERPINE1,HRG,APOA1,ITGAV,A1BG,KLKB1,KNG1,ITIH4,ALB,PLG,CLU,A2M,HBB,SERPINC1,GIG25,TF,SERPINA1 | |  |
| HSA-381426 | Regulation of Insulin-like Growth Factor (IGF) transport and uptake by Insulin-like Growth Factor Binding Proteins (IGFBPs) | | 10 | | 124 | | 1.5 | | 5.57E-10 | | APOA1,C3,CP,KNG1,ALB,PLG,SERPINC1,TF,SERPINA1,IGFALS | |  |
| HSA-8957275 | Post-translational protein phosphorylation | | 8 | | 107 | | 1.47 | | 1.73E-07 | | APOA1,C3,CP,KNG1,ALB,SERPINC1,TF,SERPINA1 | |  |
| HSA-2168880 | Scavenging of heme from plasma | | 5 | | 13 | | 2.18 | | 2.27E-07 | | APOA1,HPX,ALB,HBB,HP | |  |
| HSA-977606 | Regulation of Complement cascade | | 6 | | 47 | | 1.7 | | 1.14E-06 | | VTN,C3,CFP,C9,CLU,CFH | |  |
| HSA-140837 | Intrinsic Pathway of Fibrin Clot Formation | | 5 | | 23 | | 1.93 | | 1.99E-06 | | F9,KLKB1,KNG1,A2M,SERPINC1 | |  |
| HSA-174824 | Plasma lipoprotein assembly, remodeling, and clearance | | 6 | | 71 | | 1.52 | | 7.09E-06 | | APOC3,APOA1,LCAT,ALB,A2M,APOA4 | |  |
| HSA-8963899 | Plasma lipoprotein remodeling | | 5 | | 32 | | 1.79 | | 7.09E-06 | | APOC3,APOA1,LCAT,ALB,APOA4 | |  |
| HSA-8964058 | HDL remodeling | | 4 | | 10 | | 2.19 | | 7.09E-06 | | APOC3,APOA1,LCAT,ALB | |  |
| HSA-168249 | Innate Immune System | | 14 | | 1025 | | 0.73 | | 2.66E-05 | | VTN,TTR,C3,CFP,ITGAV,A1BG,C9,CLU,HBB,HP,CFH,GIG25,DSN1,SERPINA1 | |  |
| **(C) EC and Hy** | | | | | | | | | | | | | |
| **#term ID** | | **term description** | | **observed gene count** | | **background gene count** | | **strength** | | **false discovery rate** | | **matching proteins in your network (labels)** | |
|  | |  | |  | |  | |  | |  | |  | |
| hsa04610 | | Complement and coagulation cascades | | 11 | | 82 | | 1.81 | | 1.40E-14 | | F9,C3,KLKB1,KNG1,F2,PLG,CLU,A2M,CFH,SERPINC1,SERPINA1 | |
| hsa04979 | | Cholesterol metabolism | | 4 | | 48 | | 1.6 | | 0.0007 | | APOC3,APOA1,LCAT,APOA4 | |
| hsa03018 | | RNA degradation | | 3 | | 75 | | 1.28 | | 0.0389 | | EXOSC5,EXOSC7,DIS3L | |
| hsa03320 | | PPAR signaling pathway | | 3 | | 75 | | 1.28 | | 0.0389 | | APOC3,APOA1,CPT1A | |
| HSA-381426 | | Regulation of Insulin-like Growth Factor (IGF) transport and uptake by Insulin-like Growth Factor Binding Proteins (IGFBPs) | | 11 | | 124 | | 1.63 | | 6.27E-12 | | APOA1,C3,CP,KNG1,ALB,F2,PLG,SERPINC1,TF,SERPINA1,IGFALS | |
| HSA-109582 | | Hemostasis | | 16 | | 605 | | 1.1 | | 2.27E-11 | | F9,HRG,APOA1,KLKB1,KNG1,ITIH4,ALB,F2,PLG,CLU,A2M,HBB,SERPINC1,GIG25,TF,SERPINA1 | |
| HSA-76002 | | Platelet activation, signaling and aggregation | | 12 | | 260 | | 1.34 | | 9.57E-11 | | HRG,APOA1,KNG1,ITIH4,ALB,F2,PLG,CLU,A2M,GIG25,TF,SERPINA1 | |
| HSA-140837 | | Intrinsic Pathway of Fibrin Clot Formation | | 6 | | 23 | | 2.1 | | 9.47E-09 | | F9,KLKB1,KNG1,F2,A2M,SERPINC1 | |
| HSA-8957275 | | Post-translational protein phosphorylation | | 8 | | 107 | | 1.55 | | 2.76E-08 | | APOA1,C3,CP,KNG1,ALB,SERPINC1,TF,SERPINA1 | |
| HSA-2168880 | | Scavenging of heme from plasma | | 5 | | 13 | | 2.26 | | 7.08E-08 | | APOA1,HPX,ALB,HBB,HP | |
| HSA-174824 | | Plasma lipoprotein assembly, remodeling, and clearance | | 6 | | 71 | | 1.61 | | 2.63E-06 | | APOC3,APOA1,LCAT,ALB,A2M,APOA4 | |
| HSA-8963899 | | Plasma lipoprotein remodeling | | 5 | | 32 | | 1.87 | | 2.63E-06 | | APOC3,APOA1,LCAT,ALB,APOA4 | |
| HSA-8964058 | | HDL remodeling | | 4 | | 10 | | 2.28 | | 2.94E-06 | | APOC3,APOA1,LCAT,ALB | |
| HSA-392499 | | Metabolism of proteins | | 18 | | 1977 | | 0.64 | | 4.30E-06 | | F9,EXOSC5,APOA1,TTR,C3,CFP,CP,KNG1,EXOSC7,EIF4H,ALB,F2,PLG,APOA4,SERPINC1,TF,SERPINA1,IGFALS | |
| HSA-977606 | | Regulation of Complement cascade | | 5 | | 47 | | 1.71 | | 1.09E-05 | | C3,CFP,F2,CLU,CFH | |
| HSA-8963898 | | Plasma lipoprotein assembly | | 4 | | 19 | | 2 | | 1.92E-05 | | APOC3,APOA1,A2M,APOA4 | |
